# WNK1 links ionic signals to microtubule dynamics to control pyrin inflammasome assembly

**DOI:** 10.1126/sciadv.aeh9198

**Published:** 2026-10-16

**Authors:** Genevieve Lewis, Lindsey Mayes-Hopfinger, Md Kaisar Ali, Jian Xie, Chou-Long Huang, Hanping Feng, Teresa Fernandes-Alnemri, Emad S. Alnemri

**Affiliations:** ^1^ Department of Biochemistry and Molecular Biology, Thomas Jefferson University, Philadelphia, PA 19107, USA.; ^2^ Department of Medicine, Division of Nephrology, University of Iowa Carver College of Medicine, Iowa City, IA 52242-1081, USA.; ^3^ Department of Microbial Pathogenesis, University of Maryland at Baltimore, Baltimore, MD 21201, USA.; ^4^ Sidney Kimmel Comprehensive Cancer Center, Thomas Jefferson University, Philadelphia, PA 19107, USA.

## Abstract

WNK1 [with no lysine (K)] is a chloride- and potassium-sensitive kinase that regulates cellular ion homeostasis. Although WNK1 negatively regulates the NLRP3 and AIM2 inflammasomes, its role in pyrin inflammasome activation has remained unknown. Here, we identify WNK1 as a critical regulator of pyrin inflammasome assembly. In macrophages, genetic or pharmacologic inhibition of WNK1 suppresses pyrin activation without affecting pyrin dephosphorylation, demonstrating that WNK1 acts downstream of the canonical licensing step. This function occurs largely independently of the canonical SPAK/OXSR1 pathway. Mechanistically, WNK1 regulates microtubule dynamics: Its inhibition stabilizes microtubules, reduces plus-end growth, and impairs pyrin inflammasome assembly. Extracellular ionic conditions bidirectionally regulate this pathway, with hypotonic stress enhancing WNK1 activity and pyrin activation, whereas elevated extracellular potassium chloride suppresses WNK1 activity and inhibits pyrin assembly independently of osmotic stress. Together, these findings define an ion-sensitive WNK1-microtubule axis linking ionic signals to cytoskeletal dynamics to control pyrin inflammasome assembly and explain how WNK1 exerts opposing effects on distinct inflammasome pathways.

## INTRODUCTION

Inflammasomes are cytosolic signaling complexes that activate caspase-1, drive interleukin-1β (IL-1β) and IL-18 maturation, and induce pyroptotic cell death in response to infection and tissue damage (*1*–*4*). Among them, the pyrin inflammasome functions as a sensor of bacterial toxins that inactivate Rho guanosine triphosphatases (GTPases), a family of small guanosine 5′-triphosphate (GTP)–binding proteins that regulate cytoskeletal dynamics and innate immune functions (*5*, *6*). *Clostridioides difficile* toxin B (TcdB) and related RhoA-glucosylating toxins trigger pyrin activation by disrupting RhoA-dependent signaling, leading to loss of inhibitory phosphorylation and assembly of pyrin with the adaptor ASC and caspase-1 (*7*, *8*). Pyrin activation thus represents a critical mechanism by which macrophages detect pathogen-induced perturbations of RhoA-regulated cellular processes, including cytoskeletal organization. While this pathway has been well defined at the level of RhoA signaling, the downstream cellular processes that enable productive inflammasome assembly and the broader biophysical context in which pyrin activation occurs remain poorly understood.

Microtubule dynamics have emerged as a key determinant of pyrin inflammasome activation (*7*). Both microtubule depolymerizing and stabilizing agents, including colchicine and paclitaxel, suppress pyrin activation (*7*), indicating that dynamic microtubule remodeling is required for efficient assembly of the pyrin-ASC complex. These observations support a model in which pyrin activation depends on a permissive cytoskeletal state that allows trafficking and spatial organization of inflammasome components. However, the upstream signaling pathways that regulate microtubule dynamics during inflammasome activation are largely unknown. Defining these pathways is particularly important, as dysregulated pyrin activation underlies autoinflammatory disorders such as Familial Mediterranean Fever and pyrin-associated autoinflammation with neutrophilic dermatosis (*7*, *9*, *10*).

Cellular ionic composition is a fundamental determinant of both cytoskeletal organization and innate immune signaling (*11*–*13*). Changes in intracellular potassium and chloride concentrations influence cytoskeletal dynamics, intracellular organization, and signaling processes (*11*–*13*). However, how these ionic cues are integrated into inflammasome regulation remains incompletely defined.

The serine/threonine kinase WNK1 [with no lysine (K)] is a central sensor of intracellular chloride and potassium that regulates ion transport and cell volume homeostasis (*14*–*16*). Canonically, WNK1 activates the downstream kinases OXSR1 and SPAK to control cation-chloride cotransporters and maintain ionic balance (*17*). Consistent with this role, WNK1 also regulates innate immune signaling, including activation of the NLRP3 inflammasome through control of intracellular chloride homeostasis (*18*).

In addition to these well-established functions, WNK1 has emerged as a regulator of cytoskeletal organization, vesicular trafficking, and cell migration (*19*–*23*). In immune cells, WNK1-dependent pathways control macropinocytosis, efferocytosis, and actin remodeling (*20*, *24*, *25*), and recent work further links WNK1 to the integration of ionic and biophysical cues that govern microtubule-dependent processes (*26*). Together, these findings suggest that WNK1 functions as a central coordinator of ionic and biophysical signals that regulate cytoskeletal organization and immune signaling.

Here, we identify WNK1 as a previously unrecognized regulator of pyrin inflammasome activation. We show that genetic deletion or pharmacologic inhibition of WNK1 suppresses pyrin activation in macrophages stimulated with TcdB, without affecting pyrin dephosphorylation, indicating that WNK1 functions downstream of the canonical licensing step. This regulation occurs largely independently of the SPAK/OXSR1 pathway, revealing a noncanonical role for WNK1 in inflammasome signaling. Mechanistically, we demonstrate that WNK1 controls microtubule dynamics: Its inhibition stabilizes microtubules and reduces microtubule growth, thereby impairing pyrin inflammasome assembly. Furthermore, extracellular ionic conditions bidirectionally regulate this pathway, with hypotonic stress enhancing WNK1 activity and pyrin activation, whereas elevated extracellular KCl suppresses WNK1 activity and inhibits pyrin assembly independently of osmotic stress.

Together, our findings define a WNK1-microtubule axis that links cellular ionic states to pyrin inflammasome assembly. This work reveals a previously unappreciated mechanism by which cellular ionic states are coupled to microtubule dynamics to regulate innate immune signaling and establishes WNK1 as a key integrator of ionic and cytoskeletal cues governing pyrin inflammasome assembly.

## RESULTS

### WNK1 is required for pyrin inflammasome activation

We previously demonstrated that WNK1 functions as a negative regulator of the NLRP3 inflammasome, restraining inflammasome activation by maintaining intracellular Cl^−^ and K^+^ homeostasis through the downstream kinases OXSR1 and SPAK (*18*). Accordingly, inhibition or genetic deletion of WNK1 in macrophages enhances NLRP3 inflammasome activation, leading to increased pyroptosis and elevated IL-1β production.

To determine whether WNK1 similarly regulates other inflammasomes, we examined its role in pyrin inflammasome activation. Bone marrow–derived macrophages (BMDMs) were stimulated with TcdB, a prototypical RhoA-inactivating toxin that activates the pyrin inflammasome (*7*, *8*). Wild-type (WT) BMDMs exhibited robust pyrin inflammasome activation, as assessed by propidium iodide (PI) uptake, caspase-1 p20 processing, gasdermin D (GSDMD) cleavage, ASC oligomerization, and IL-1β secretion (Fig. 1, A to F). In contrast, BMDMs lacking WNK1 (WNK1-KO) displayed markedly reduced pyrin inflammasome activation across all readouts, despite comparable expression levels of pyrin, ASC, and caspase-1 (Fig. 1, A to F). Similar inhibition of pyrin inflammasome activation was observed following both short (2 hours) and prolonged (8 hours) lipopolysaccharide (LPS) priming, demonstrating that the requirement for WNK1 is independent of the duration of the priming step (fig. S1). These findings identify WNK1 as a critical positive regulator required for efficient pyrin inflammasome activation in response to TcdB.

**Fig. 1.**
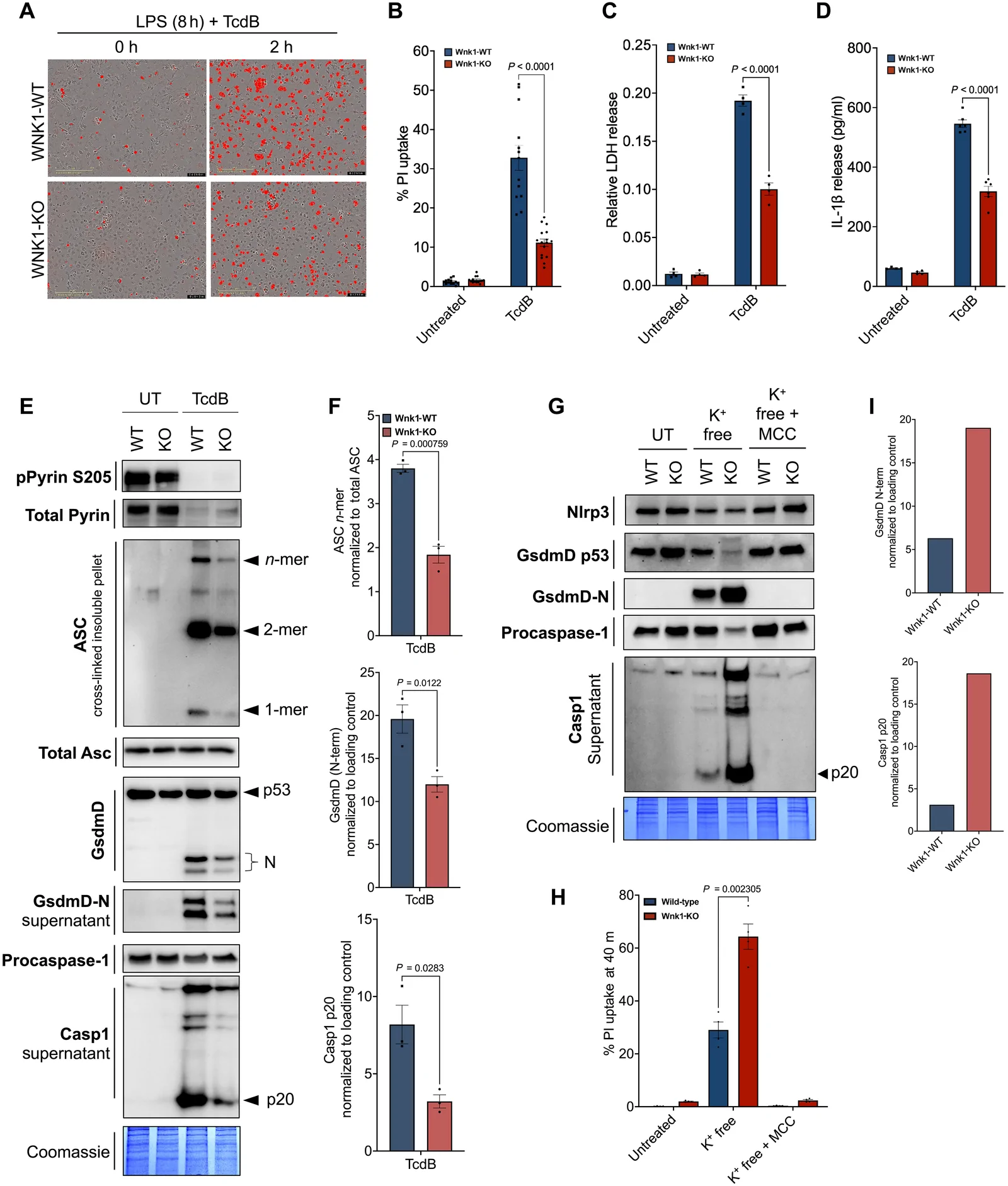
Genetic deletion of WNK1 differentially regulates pyrin and NLRP3 inflammasomes. (**A**) Representative IncuCyte S3 images of PI uptake in LPS-primed primary Wnk1^+/+^ LysMCre^+^ (Wnk1-WT) and Wnk1^fl/fl^ LysMCre^+^ (Wnk1-KO) BMDMs stimulated with TcdB (5 ng ml^−1^) in the presence of the NLRP3 inhibitor MCC950 (5 μM) and imaged for 3.5 hours (h). (**B** to **D**) Quantification of PI uptake (B), LDH release (C), and IL-1β release (D) into the culture supernatant from the cells in (A) at 3.5 hours poststimulation. (**E**) Immunoblot analysis of pyrin inflammasome activation in cells treated as in (A). Cell lysates were probed for pPyrin (S205), total pyrin, GSDMD, procaspase-1, and total ASC. ASC oligomers were detected in BS^3^–cross-linked NP-40–insoluble pellets. Culture supernatants were analyzed for caspase-1 p20 and GSDMD-N. (**F**) Densitometric quantification of ASC oligomers, GSDMD-N, and caspase-1 p20 from four independent experiments as in (A). UT, untreated. (**G**) Immunoblot analysis of NLRP3 inflammasome activation in primary Wnk1-WT and Wnk1-KO BMDMs stimulated with LPS in potassium-free medium (to induce NLRP3 activation) in the absence or presence of MCC950 (5 μM) for 40 min. Lysates were probed for NLRP3, GSDMD, GSDMD-N, and procaspase-1; supernatants were analyzed for caspase-1 p20. (**H**) Quantification of PI uptake from the cells in (G) at 40 min. (**I**) Densitometric quantification of GSDMD-N and caspase-1 p20 from the immunoblots shown in (G). Coomassie-stained gels of cell lysates in (E) and (G) (bottom) serve as loading controls. Data are representative of at least three independent experiments and are presented as means ± SEM. Statistical significance was determined by two-tailed unpaired *t* test.

Consistent with our previous findings (*18*), WNK1 deficiency enhanced NLRP3 inflammasome activation in response to potassium efflux or nigericin (Fig. 1, G to I, and fig. S2, A to E). Thus, WNK1 exerts opposing effects on distinct inflammasomes, functioning as a suppressor of NLRP3 activation while serving as a permissive factor for pyrin inflammasome activation.

### Kinase activity of WNK1 is required for pyrin inflammasome activation

To determine whether pyrin inflammasome activation requires WNK1 kinase activity, we treated WT BMDMs with the selective WNK1 inhibitor WNK463 (*27*) (1.5 μM; 30 min pretreatment) before stimulation with TcdB. Pharmacologic inhibition of WNK1 phenocopied genetic deletion, resulting in marked suppression of pyrin inflammasome activation, as evidenced by reduced PI uptake, lactate dehydrogenase (LDH) release, ASC oligomerization, GSDMD cleavage, and caspase-1 p20 processing (Fig. 2, A to F). Similar suppression of inflammasome activation was observed in BMDMs lacking both NLRP3 and AIM2, demonstrating that the effect of WNK1 inhibition is specific to the pyrin inflammasome and not secondary to altered NLRP3 or AIM2 signaling (Fig. 2, G to L).

**Fig. 2.**
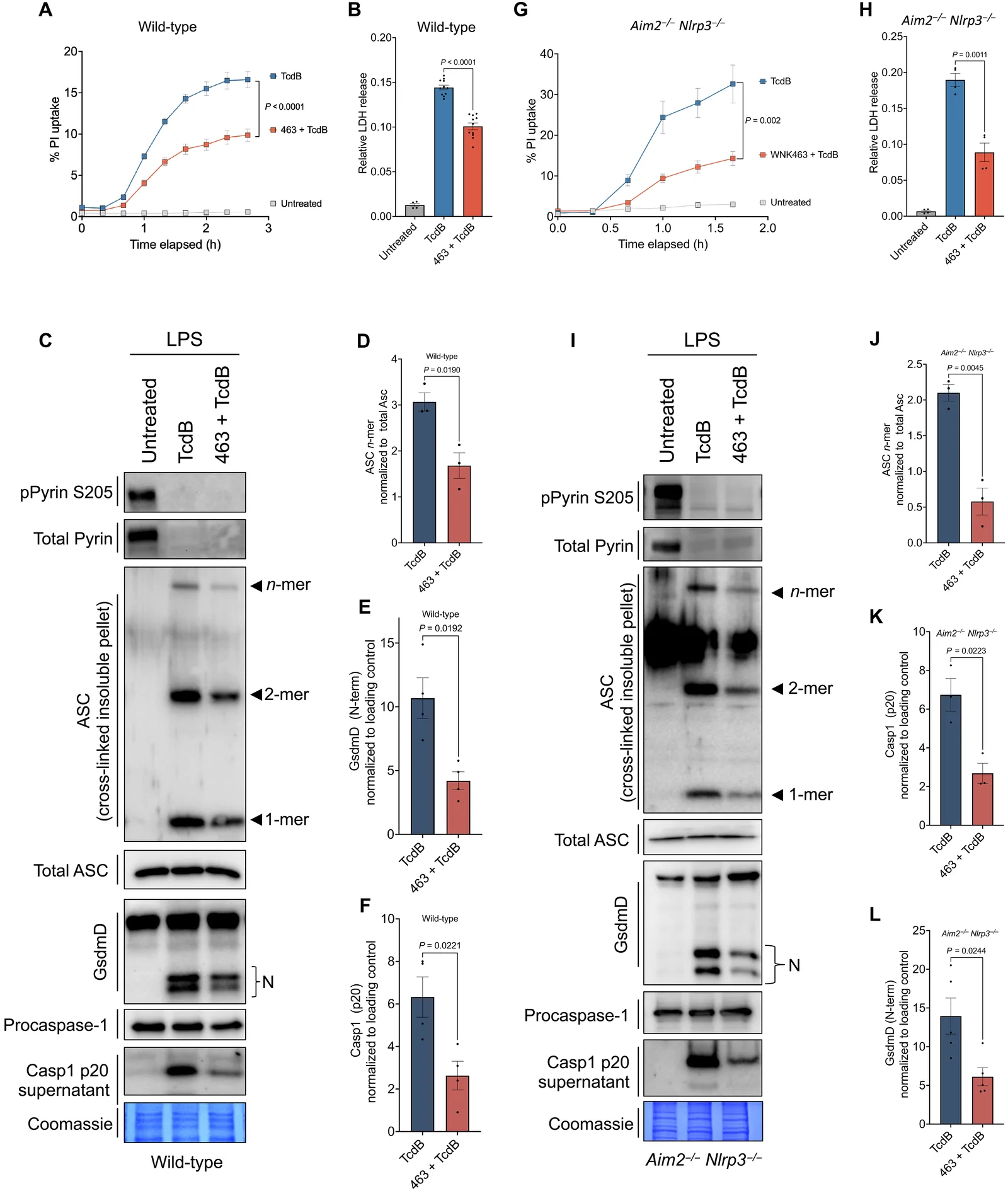
Pharmacologic inhibition of WNK1 suppresses pyrin inflammasome activation. (**A** and **B**) LPS-primed primary WT BMDMs were stimulated with TcdB (5 ng ml^−1^) in the presence of the NLRP3 inhibitor MCC950 (5 μM), with or without the WNK1 inhibitor WNK463 (1.5 μM). (A) Quantification of PI uptake over time. (B) Relative LDH release measured in culture supernatants from independent experiments. (**C** to **F**) Immunoblot analysis of pyrin inflammasome activation in WT BMDMs treated as in (A). Shown are pPyrin (S205), total pyrin, ASC oligomers in BS^3^–cross-linked NP-40–insoluble pellets, total ASC, GSDMD, procaspase-1, and caspase-1 p20 in supernatants. Coomassie staining of cell lysates is shown as a loading control. [(D) to (F)] Densitometric quantification of ASC oligomers (D), GSDMD cleavage (N-terminal fragment) (E), and caspase-1 p20 (F) from independent experiments. (**G** and **H**) LPS-primed Aim2^−/−^ Nlrp3^−/−^ BMDMs were treated as in (A). (G) Quantification of PI uptake over time. (H) Relative LDH release in culture supernatants from three independent experiments. (**I**) Immunoblot analysis of pyrin inflammasome activation in Aim2^−/−^ Nlrp3^−/−^ BMDMs treated as in (G), including pPyrin (S205), total pyrin, ASC oligomers, total ASC, GSDMD, procaspase-1, and caspase-1 p20 in supernatants. (**J** to **L**) Densitometric quantification of ASC oligomers, GSDMD cleavage, and caspase-1 p20 from independent experiments. PI uptake curves shown in different panels were obtained from independent experiments and are intended for comparison only within each experiment. Data are representative of at least three independent experiments and are presented as means ± SEM. Statistical significance was determined by two-tailed unpaired *t* test.

To determine whether WNK1 regulates pyrin inflammasome assembly independently of downstream caspase activation or necroptotic signaling, we next analyzed Ripk3^−/−^ and Mlkl^−/−^ BMDMs in the presence or absence of the pan-caspase inhibitor emricasan (fig. S3). Pyrin-induced ASC oligomerization, as well as its suppression by WNK463, was preserved in Ripk3^−/−^ and Mlkl^−/−^ macrophages and was unaffected by caspase inhibition. These findings indicate that WNK1 regulates pyrin inflammasome assembly at the level of ASC oligomerization independently of downstream caspase activity and necroptotic signaling. These data place WNK1 function upstream of terminal inflammasome effector pathways and define ASC oligomerization as a primary point of regulation.

To determine whether this requirement for WNK1 kinase activity is generalizable across pyrin-activating stimuli, we examined responses to *C. difficile* toxin A (TcdA) (*7*). WNK463 suppressed TcdA-induced pyrin activation across a range of toxin concentrations and priming conditions, as reflected by reduced PI uptake, GSDMD cleavage, and caspase-1 processing (fig. S4). Consistent with our previous findings (*18*), WNK1 inhibition enhanced NLRP3 inflammasome activation in response to adenosine 5′-triphosphate (ATP; fig. S2F). Together, these data demonstrate that WNK1 kinase activity is required for pyrin inflammasome activation and differentially regulates pyrin and NLRP3 inflammasomes.

### The canonical SPAK/OXSR1 pathway is dispensable for WNK1-dependent pyrin activation

WNK1 is best characterized as an upstream serine/threonine kinase that signals through the related kinases OXSR1 and SPAK to regulate ion transport, cell volume homeostasis, and downstream inflammatory responses (*17*, *18*, *28*). In macrophages, we previously demonstrated that this canonical WNK1-SPAK/OXSR1 signaling cascade restrains NLRP3 inflammasome activation by maintaining intracellular Cl^−^ and K^+^ homeostasis (*18*).

To determine whether WNK1 regulates pyrin inflammasome activation through this established SPAK/OXSR1 signaling cascade, we first examined BMDMs lacking both downstream kinases (SPAK/OXSR1-dKO). Pharmacologic inhibition of WNK1 with WNK463 suppressed pyrin inflammasome activation to a similar extent in SPAK/OXSR1-dKO and WT BMDMs, as assessed by PI uptake, GSDMD cleavage, and caspase-1 processing (Fig. 3, A to E). In contrast, loss of OXSR1 and SPAK enhanced NLRP3 inflammasome activation, consistent with our previous findings (Fig. 3, F to H). These results indicate that WNK1 regulates pyrin inflammasome activation largely independently of the canonical SPAK/OXSR1 pathway, in contrast to its established role in restraining NLRP3 activation through these downstream kinases (*18*, *28*).

**Fig. 3.**
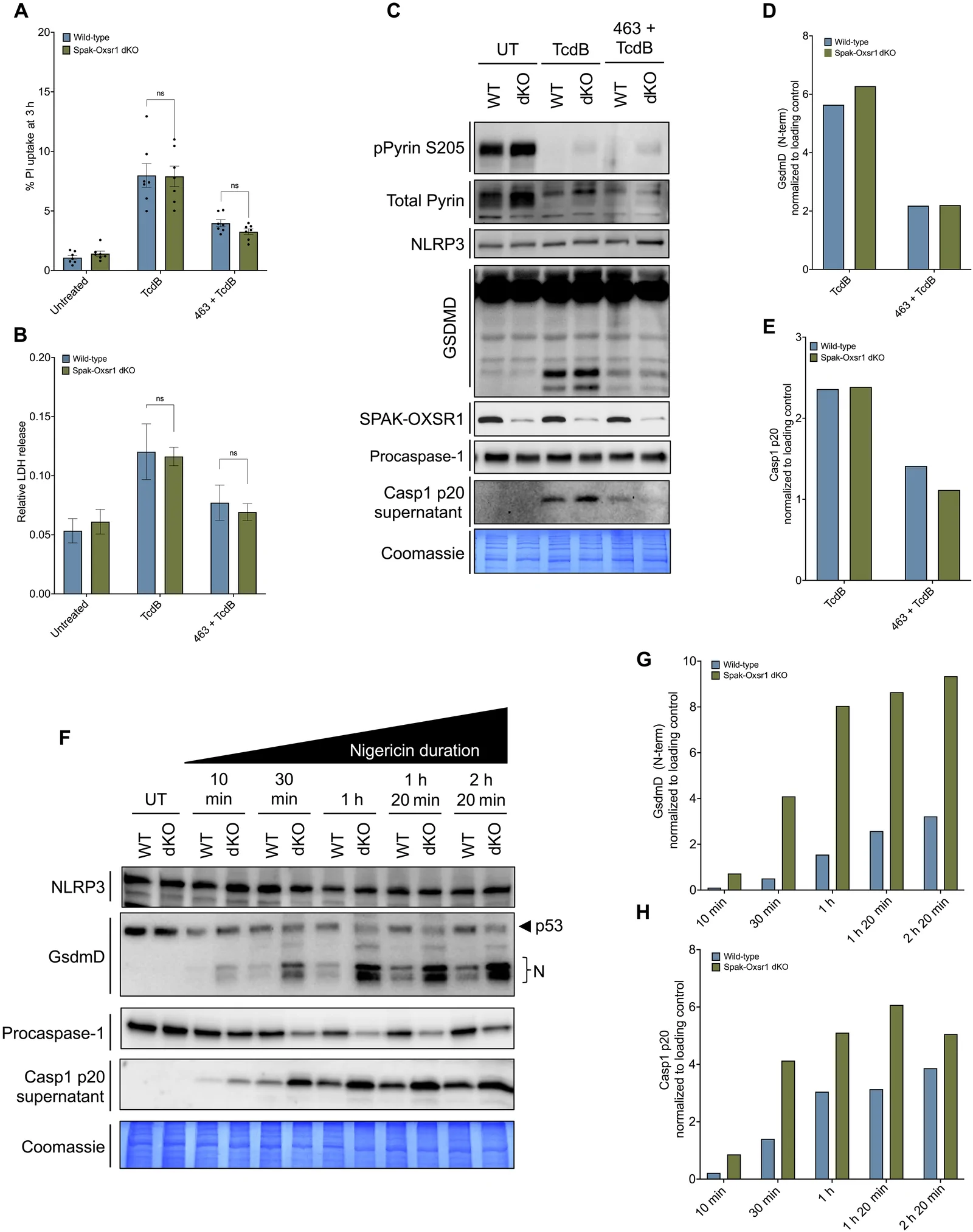
SPAK and OXSR1 are dispensable for WNK1-dependent pyrin inflammasome activation. (**A** and **B**) LPS-primed (500 ng ml^−1^, 5 hours) primary WT (LysMCre^+^) and Spak^fl/fl^ Oxsr1^fl/fl^ LysMCre^+^ (Spak/Oxsr1 conditional dKO) BMDMs were pretreated with MCC950 (5 μM) in the presence or absence of the WNK1 inhibitor WNK463 (1.5 μM) for 15 min, followed by stimulation with TcdB (5 ng ml^−1^) for 3 hours. (A) Quantification of PI uptake at the indicated time point. (B) Relative LDH release in culture supernatants. (**C**) Immunoblot analysis of pyrin inflammasome activation in WT and Spak/Oxsr1 conditional dKO BMDMs treated as in (A). Shown are pPyrin (S205), total pyrin, GSDMD, procaspase-1, and Spak/OXSR1 in cell lysates as well as caspase-1 p20 in supernatants. Coomassie staining is shown as a loading control. (**D** and **E**) Densitometric quantification of GSDMD cleavage (N-terminal fragment) (D) and caspase-1 p20 (E) from the immunoblots shown in (C). (**F**) LPS-primed (500 ng ml^−1^, 3 hours) WT and Spak/Oxsr1 conditional dKO BMDMs were stimulated with nigericin (10 μM) for the indicated times. Immunoblot analysis of NLRP3 inflammasome activation showing NLRP3, GSDMD, procaspase-1 in cell lysates, and caspase-1 p20 in supernatants. Coomassie staining is shown as a loading control. Data are representative of at least three independent experiments. (**G** and **H**) Densitometric quantification of GSDMD cleavage (N-terminal fragment) (G) and caspase-1 p20 (H) from the immunoblots shown in (F). Data are representative of at least three independent experiments and are presented as means ± SEM. Statistical significance was determined by two-tailed unpaired *t* test.

To further exclude involvement of SPAK/OXSR1 signaling, we next examined BMDMs expressing a constitutively active SPAK (SPAK^CA^) mutant (*29*) in either WT or WNK1-deficient backgrounds. Consistent with the results obtained in SPAK/OXSR1-dKO macrophages, expression of SPAK^CA^ failed to restore pyrin inflammasome activation in WNK1-deficient BMDMs, as measured by ASC oligomerization, GSDMD cleavage, and caspase-1 processing (Fig. 4, A to F). Moreover, pharmacologic inhibition of WNK1 suppressed pyrin inflammasome activation in SPAK^CA^-expressing WT BMDMs to a similar extent as in control cells (Fig. 4, G to K), indicating that enforced SPAK signaling is insufficient to bypass the requirement for WNK1 in pyrin activation.

**Fig. 4.**
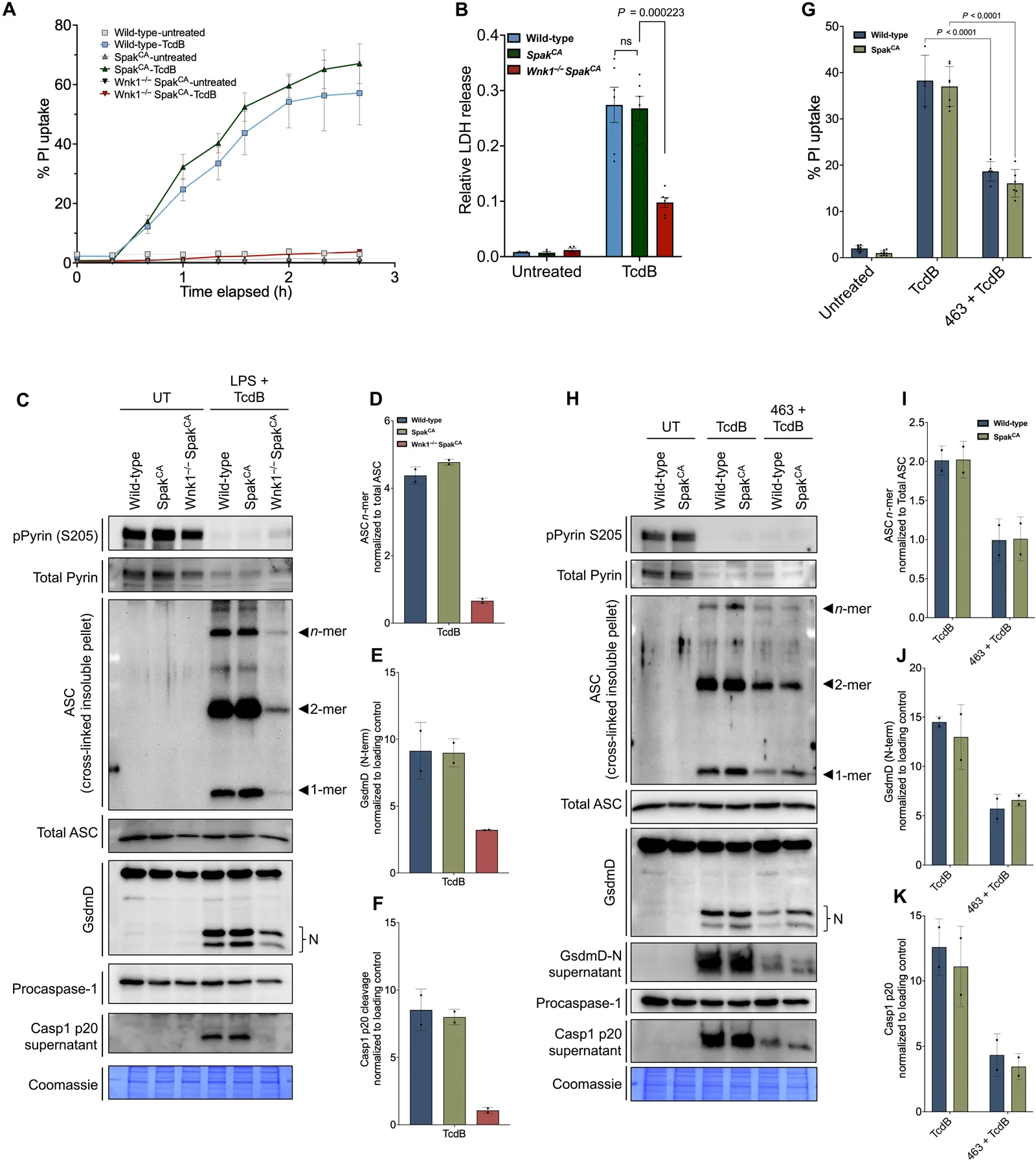
Constitutive SPAK signaling does not restore pyrin inflammasome activation in the absence of WNK1. (**A** and **B**) LPS-primed (500 ng ml^−1^, 6 hours) primary WT, Spak^CA^, and Wnk1^−/−^ Spak^CA^ BMDMs were pretreated with MCC950 (5 μM) for 15 min and stimulated with TcdB (5 ng ml^−1^). (A) Quantification of PI uptake over time, measured by IncuCyte live-cell imaging. (B) Relative LDH release in culture supernatants. (**C**) Immunoblot analysis of pyrin inflammasome activation in WT, Spak^CA^, and Wnk1^−/−^ Spak^CA^ BMDMs treated as in (A). Shown are pPyrin (S205), total pyrin, ASC oligomers (BS^3^–cross-linked NP-40–insoluble fraction), total ASC, GSDMD, procaspase-1, and caspase-1 p20 in supernatants. Coomassie staining is shown as a loading control. (**D** to **F**) Densitometric quantification of ASC oligomers (D), GSDMD cleavage (N-terminal fragment) (E), and caspase-1 p20 (F) from two independent experiments as in (C). (**G**) LPS-primed (500 ng ml^−1^, 6 hours) WT and Spak^CA^ BMDMs were pretreated with MCC950 (5 μM) and WNK463 (1.5 μM) and stimulated with TcdB (5 ng ml^−1^) for 4 hours. Quantification of PI uptake at 4 hours after TcdB stimulation. (**H**) Immunoblot analysis of WT and Spak^CA^ BMDMs treated as in (G), showing pPyrin (S205), total pyrin, ASC oligomers, total ASC, GSDMD, procaspase-1, and caspase-1 p20 in supernatants. Coomassie staining is shown as a loading control. (**I** to **K**) Densitometric quantification of ASC oligomers (I), GSDMD cleavage (J), and caspase-1 p20 (K) from two independent experiments as in (H). Data are representative of at least three independent experiments and are presented as means ± SEM. Statistical significance was determined by two-tailed unpaired *t* test. ns, not significant.

In contrast, SPAK^CA^ expression markedly suppressed NLRP3 inflammasome activation in both WT and WNK1-deficient macrophages, as evidenced by reduced PI uptake, GSDMD cleavage, and caspase-1 processing under potassium efflux conditions (fig. S5). Thus, whereas SPAK mediates the inhibitory effect of WNK1 on NLRP3, it does not contribute to WNK1-dependent regulation of the pyrin inflammasome. Collectively, these findings demonstrate that WNK1 regulates pyrin inflammasome activation largely independently of its canonical SPAK/OXSR1 ion-regulatory pathway, supporting the existence of an additional downstream mechanism.

### WNK1 regulates pyrin inflammasome activation downstream of pyrin dephosphorylation

Following cytosolic delivery, TcdB inactivates Rho GTPases, leading to loss of PKN1/2 activity and release of pyrin from inhibitory phosphorylation and 14-3-3 binding (*7*, *8*, *30*). To define the step at which WNK1 regulates pyrin inflammasome activation, we first asked whether WNK1 inhibition interferes with toxin entry or upstream RhoA-PKN signaling.

Because cytosolic delivery of TcdB requires endosomal acidification to release its glucosyltransferase domain (*31*, *32*), we examined the effect of WNK1 inhibition on toxin processing and RhoA inactivation. Inhibition of WNK1 with WNK463 did not impair TcdB-induced RhoA inactivation or pyrin dephosphorylation, indicating that toxin entry and upstream RhoA-PKN signaling remain intact (fig. S6, A to E). In contrast, pretreatment with bafilomycin A1, which blocks endosomal acidification and toxin entry, prevented pyrin dephosphorylation and strongly suppressed caspase-1 processing, whereas bafilomycin added after TcdB exposure had a markedly reduced effect (fig. S6, C to E).

We next tested whether bypassing the PKN-dependent inhibitory checkpoint could restore pyrin activation in the absence of WNK1. Pharmacologic inhibition of PKN1/2, which normally maintain pyrin in a phosphorylated and inactive state (*30*), enhanced TcdB-induced pyrin activation in WT macrophages. However, PKN1/2 inhibition failed to restore caspase-1 activation or GSDMD cleavage in WNK1-deficient macrophages (fig. S7, A to D). Notably, although PKN1/2 inhibition increased pyrin activation in both WT and WNK1-deficient cells, the magnitude of activation remained substantially reduced in WNK1-deficient macrophages under all conditions. Together, these findings demonstrate that WNK1 is not required for toxin entry, RhoA inactivation, or PKN1/2-dependent pyrin dephosphorylation but instead functions downstream of this licensing step to enable productive pyrin inflammasome assembly.

### WNK1 regulates microtubule stability

Because pyrin licensing was intact but inflammasome assembly remained defective in the absence of WNK1 and because pyrin activation depends on an intact microtubule cytoskeleton (*7*), we next examined whether WNK1 regulates pyrin inflammasome activation through effects on microtubule stability. To determine whether WNK1 influences microtubule stability at the level of tubulin polymerization, we performed biochemical tubulin polymerization assays (*33*). Immortalized Nlrp3^−/−^ macrophages were treated with the WNK1 inhibitor WNK463, paclitaxel, or colchicine, followed by separation of soluble and polymerized tubulin fractions. Whereas control cells exhibited a balanced distribution of α-tubulin between soluble and pellet fractions, inhibition of WNK1 notably shifted tubulin toward the polymerized fraction, indicating increased microtubule stability (Fig. 5, A and B). As expected, paclitaxel induced near-complete tubulin polymerization, whereas colchicine increased the soluble tubulin pool. These data demonstrate that WNK1 inhibition promotes tubulin polymerization and microtubule stabilization.

**Fig. 5.**
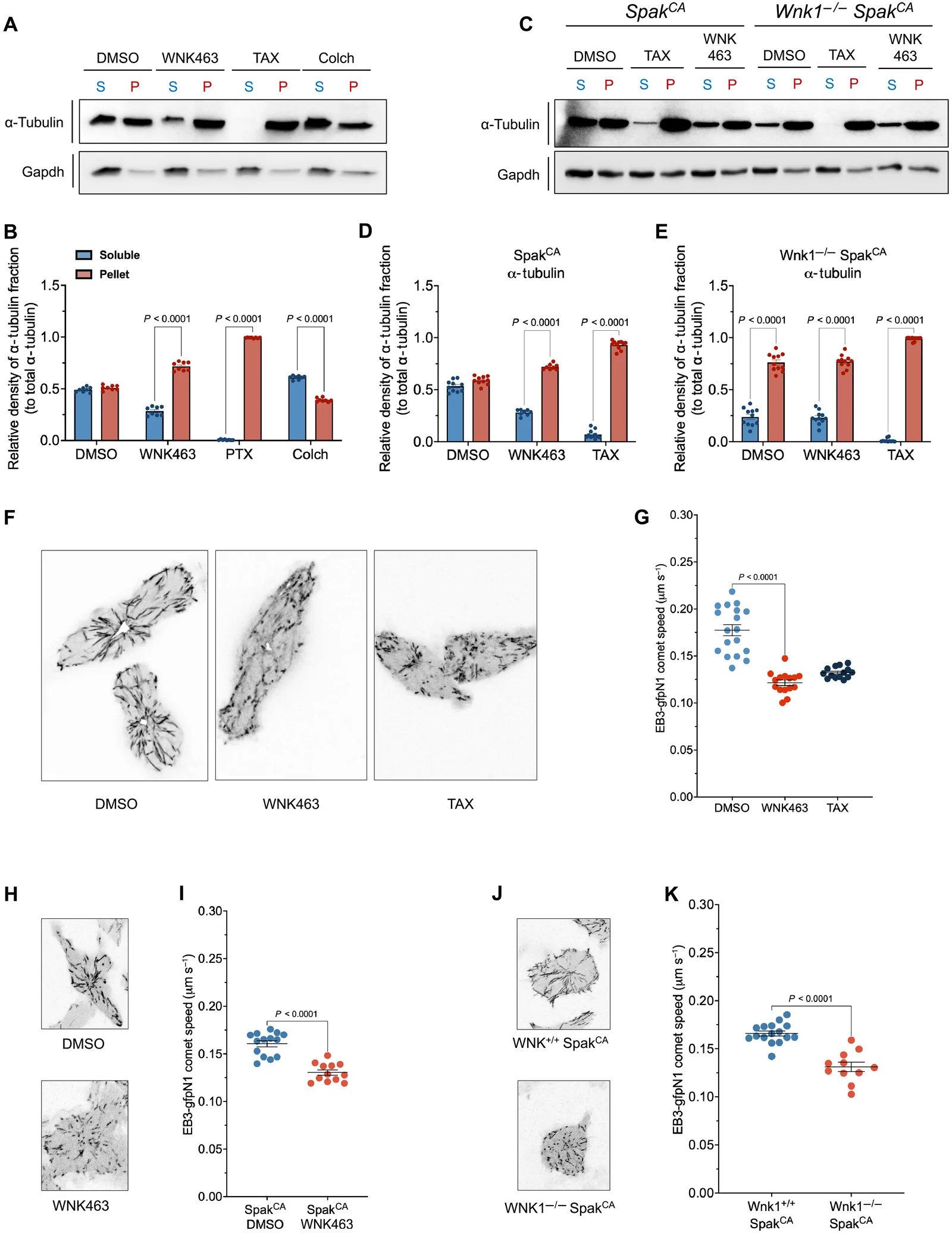
WNK1 regulates microtubule stability and dynamics independently of SPAK signaling. (**A**) Immunoblots of α-tubulin in soluble (S) and polymerized (P) fractions from immortalized Nlrp3^−/−^ macrophages treated with dimethyl sulfoxide (DMSO), WNK463 (1.5 μM), paclitaxel (TAX; 1 μM), or colchicine (1 μM) for 45 min. Glyceraldehyde-3-phosphate dehydrogenase (GAPDH) served as a soluble fraction marker and fractionation control. (**B**) Densitometric quantification of α-tubulin distribution between soluble and polymerized fractions relative to total tubulin (S + P). Data are means ± SEM (two-tailed Student’s *t* test, *n* = 7); all *P* < 0.0001. (**C**) Immunoblots of soluble and polymerized α-tubulin fractions from immortalized SpakCA and Wnk1^−/−^ SpakCA BMDMs treated as in (A). (**D** and **E**) Quantification of α-tubulin distribution from (C). Data are means ± SEM (two-tailed Student’s *t* test, *n* = 7); all *P* < 0.0001. (**F**, **H**, and **J**) Representative temporal projections of EB3-EGFP comet trajectories acquired over 60 s in immortalized Nlrp3^−/−^ macrophages treated with DMSO, WNK463 (1 μM), or paclitaxel (100 nM) (F), DMSO- or WNK463-treated SpakCA BMDMs (H), and SpakCA or Wnk1^**−/−**^ SpakCA BMDMs (J). (**G**, **I**, and **K**) Quantification of EB3-EGFP comet velocity. Each point represents the mean comet velocity of a single cell calculated from ∼50 to 250 tracked comets. Approximately 10 to 20 cells per condition from three independent experiments were analyzed, with each cell treated as the statistical unit. Horizontal lines indicate means ± SEM; significance was determined by two-tailed Student’s *t* test. All experiments are representative of at least three independent biological replicates.

Because WNK1 classically signals through the SPAK/OXSR1 pathway (*17*), we next asked whether WNK1-dependent microtubule stabilization requires SPAK activity. Tubulin polymerization assays were therefore performed in macrophages expressing SPAK^CA^, as well as in Wnk1^−/−^ SPAK^CA^ macrophages. In both contexts, inhibition or loss of WNK1 resulted in a significant shift of α-tubulin toward the polymerized fraction compared to vehicle-treated SPAK^CA^ controls (Fig. 5, C to E). As expected, paclitaxel induced near-complete tubulin polymerization under all conditions. These data indicate that WNK1-dependent microtubule stabilization occurs largely independently of SPAK signaling, supporting a noncanonical mechanism by which WNK1 regulates the microtubule cytoskeleton.

### WNK1 regulates microtubule growth dynamics

To determine whether the increased microtubule stability observed upon WNK1 inhibition is accompanied by altered microtubule dynamics, we performed live-cell imaging of EB3-positive microtubule plus ends (*34*). Immortalized Nlrp3^−/−^ macrophages stably expressing EB3–green fluorescent protein (GFP) were treated with WNK463 and imaged by time-lapse fluorescence microscopy. Quantitative analysis revealed a marked reduction in EB3 comet speed following WNK1 inhibition, indicating reduced microtubule plus-end growth rates compared with untreated cells (Fig. 5, F and G, and movies S1 to S3).

To genetically validate this effect and assess its independence from canonical WNK signaling, we next analyzed EB3 dynamics in macrophages expressing SPAK^CA^ and in Wnk1^−/−^ SPAK^CA^ macrophages. Despite enforced SPAK activity, inhibition or loss of WNK1 resulted in a significant reduction in EB3 comet speed relative to SPAK^CA^ controls (Fig. 5, H to K). Thus, both pharmacologic inhibition and genetic deletion of WNK1 suppress microtubule growth dynamics largely independently of SPAK/OXSR1 signaling.

Together, these findings demonstrate that WNK1 promotes microtubule plus-end growth and that loss of WNK1 activity leads to a shift toward stabilized, less dynamic microtubules. This shift toward stabilized, less dynamic microtubules provides a plausible mechanistic basis for defective pyrin assembly and functionally links WNK1-dependent microtubule dynamics to inflammasome competence.

### WNK1 inhibition phenocopies pharmacologic microtubule perturbation at the level of pyrin inflammasome assembly

Following pyrin dephosphorylation, productive inflammasome activation requires microtubule-dependent trafficking and assembly of pyrin, ASC, and caspase-1 (*7*). Having established that WNK1 functions downstream of pyrin dephosphorylation and regulates microtubule dynamics, we next asked whether WNK1 inhibition phenocopies the effects of pharmacologic microtubule perturbation on pyrin inflammasome activation.

In LPS-primed Aim2^−/−^ Nlrp3^−/−^ BMDMs, pharmacologic perturbation of microtubule dynamics potently suppressed TcdB-induced pyrin inflammasome activation (Fig. 6, A and B). Both stabilization and depolymerization of microtubules inhibited pyrin activation. Paclitaxel suppressed pyrin activation in a dose-dependent manner, with concentrations as low as 100 nM notably reducing ASC oligomerization and processing of GSDMD and caspase-1, whereas colchicine completely abrogated pyrin activation at low micromolar concentrations (Fig. 6B). Notably, inhibition of WNK1 with WNK463 suppressed pyrin activation to a similar extent as paclitaxel, and cotreatment with WNK463 and either paclitaxel or colchicine did not produce additional suppression (Fig. 6, A and B), consistent with WNK1 and pharmacologic microtubule perturbation acting on a common microtubule-dependent assembly pathway.

**Fig. 6.**
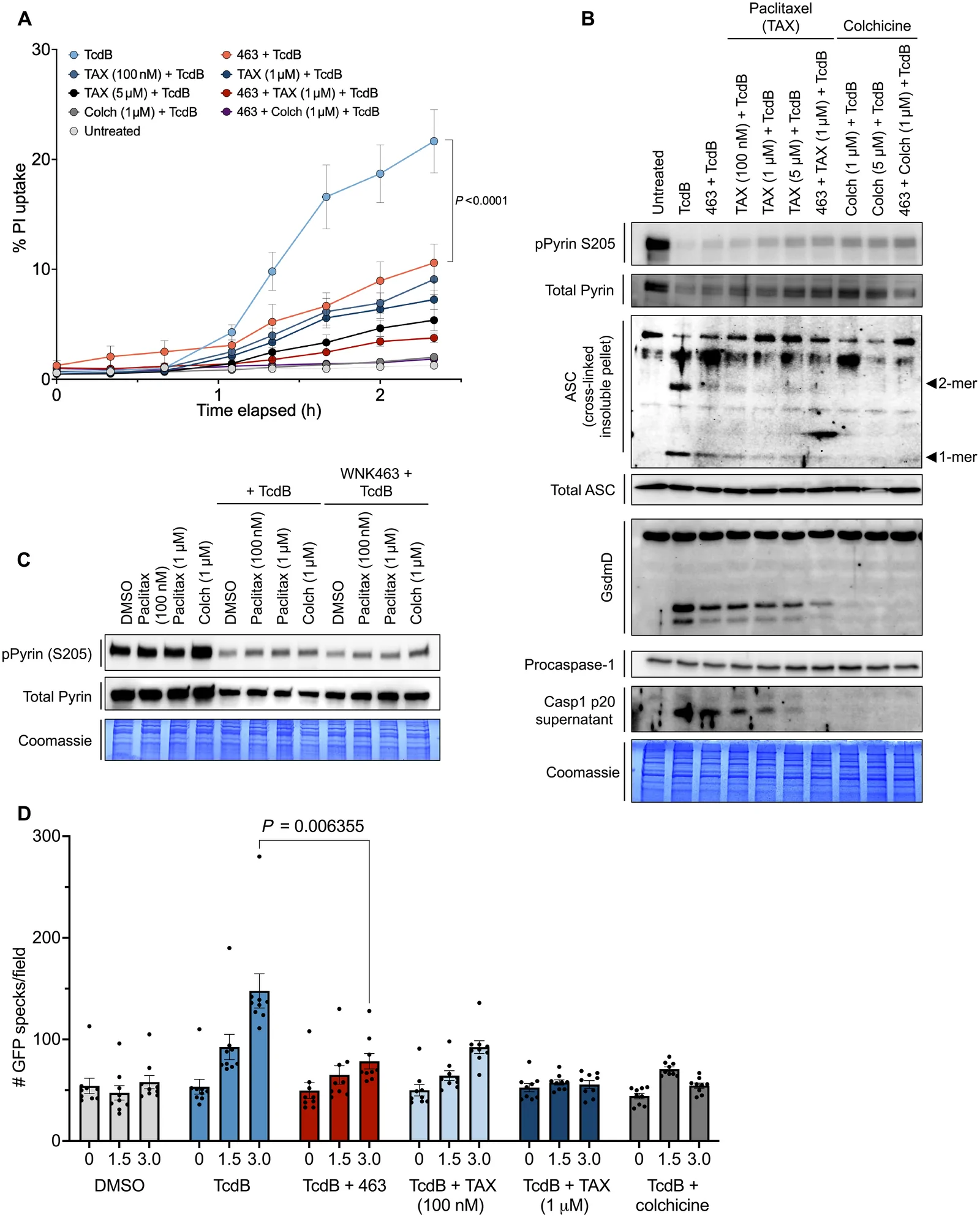
WNK1 inhibition suppresses pyrin inflammasome assembly in a manner resembling microtubule perturbation. (**A**) PI uptake in LPS-primed (500 ng ml^−1^, 8 hours) primary *Aim2*^−/−^ *Nlrp3*^−/−^ BMDMs pretreated with WNK463 (1.5 μM), paclitaxel (100 nM, 1 μM, or 5 μM), colchicine (1 μM), or the indicated combinations for 15 min, followed by stimulation with TcdB (5 ng ml^−1^). Cells were imaged using the IncuCyte S3 for 2 hours 20 min. Data are means ± SEM [two-way analysis of variance (ANOVA)]; all *P* < 0.0001. (**B**) Immunoblots of pPyrin (S205), total pyrin, GSDMD, procaspase-1, and total ASC in cell lysates; ASC oligomers in BS^3^–cross-linked NP-40–insoluble pellets; and caspase-1 p20 in culture supernatants from cells treated as in (A). (**C**) Immunoblots of pPyrin (S205) and total pyrin in immortalized *Casp1*^−/−^ macrophages stably expressing Pyrin-EGFP, pretreated with MCC950 (5 μM) with or without paclitaxel (100 nM or 1 μM), colchicine (1 μM), and/or WNK463 (1.5 μM) for 15 min before TcdB stimulation (10 ng ml^−1^). (**D**) Quantification of Pyrin-EGFP speck formation in cells from (C) at 0, 1.5, and 3 hours following TcdB stimulation. Data are means ± SEM (two-tailed Student’s *t* test, *n* = 9); *P* values at 3 hours as indicated. All data are representative of at least three independent experiments performed in duplicate or triplicate.

To directly examine pyrin inflammasome assembly independently of downstream pyroptotic events, we next used Casp1^−/−^ macrophages stably expressing pyrin–enhanced GFP (EGFP) as a fluorescent reporter of pyrin oligomerization. TcdB induced robust pyrin-EGFP speck formation, whereas both paclitaxel and colchicine markedly reduced pyrin oligomerization (Fig. 6D and fig. S8). WNK463 similarly suppressed pyrin-EGFP speck formation to an extent comparable to both paclitaxel and colchicine, despite these agents perturbing microtubule dynamics through distinct mechanisms (Fig. 6D and fig. S8).

Neither WNK1 inhibition nor pharmacologic perturbation of microtubule dynamics impaired TcdB-induced pyrin dephosphorylation (Fig. 6, B and C), indicating that their suppressive effects occur downstream of the pyrin licensing step. Together, these findings demonstrate that WNK1 inhibition phenocopies the effects of pharmacologic microtubule perturbation on pyrin inflammasome assembly, placing WNK1 within the microtubule-dependent assembly pathway downstream of pyrin dephosphorylation. The observation that both microtubule stabilization and depolymerization suppress pyrin inflammasome assembly indicates that productive assembly requires an appropriately regulated dynamic microtubule network.

### Hypotonic stress enhances pyrin inflammasome activation via WNK1, whereas high extracellular KCl suppresses pyrin activation by inhibiting WNK signaling

WNK1 kinase activity is tightly regulated by intracellular ionic composition, with both chloride and potassium acting as inhibitory ligands that suppress WNK1 autophosphorylation and downstream signaling (*35*, *36*). Given that pyrin inflammasome activation was strongly dependent on WNK1 activity in our system, we next asked whether extracellular osmotic and ionic conditions that modulate WNK1 activity influence pyrin activation.

To address this, we examined TcdB-induced pyrin inflammasome activation in LPS-treated primary Aim2^−/−^ Nlrp3^−/−^ BMDMs under isotonic and hypotonic conditions. Because SPAK is a direct substrate of WNK1, phosphorylation of SPAK was monitored solely as a biochemical indicator of WNK1 kinase activity under the various ionic conditions, rather than as evidence that SPAK mediates pyrin inflammasome activation. Exposure to hypotonic OptiMEM markedly enhanced pyrin inflammasome activation compared with isotonic medium, as assessed by increased PI uptake, ASC oligomerization, GSDMD cleavage, and caspase-1 p20 release (Fig. 7, A to G). Hypotonic conditions were accompanied by increased phosphorylation of SPAK at Ser^373^ (Fig. 7C), consistent with enhanced WNK pathway activity. These findings support the conclusion that hypotonic stress potentiates pyrin inflammasome activation through a WNK1-dependent mechanism.

**Fig. 7.**
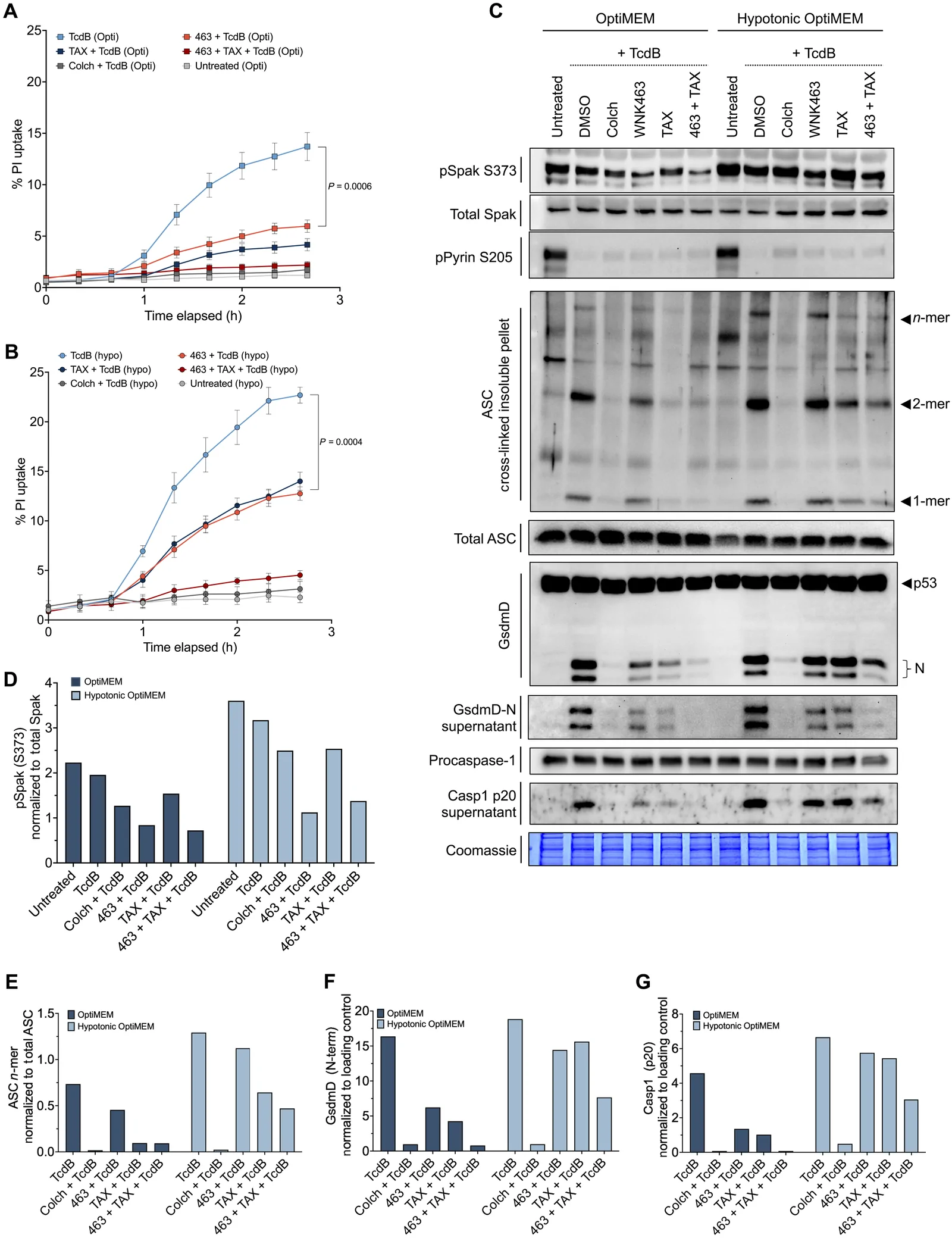
Hypotonic conditions enhance pyrin inflammasome activation and WNK1 signaling. (**A** and **B**) PI uptake of LPS-primed (500 ng ml^−1^, 8 hours) primary *Aim2*^−/−^ *Nlrp3*^−/−^ murine BMDMs pretreated with colchicine (1 μM), WNK463 (1.5 μM), or paclitaxel (1 μM) for 15 min in isotonic OptiMEM (A) or hypotonic OptiMEM (B) and subsequently stimulated with TcdB (5 ng ml^−1^). Cells were imaged using the IncuCyte S3 for 2 hours and 40 min. Data are means ± SEM (two-way ANOVA, *n* = 9). (**C**) Immunoblots of pSPAK (S373), total SPAK, pPyrin (S205), total pyrin, GSDMD, procaspase-1, and total ASC in cell lysates; ASC oligomers in BS^3^–cross-linked NP-40–insoluble pellets; and caspase-1 p20 and GSDMD-N in serum-free supernatants from cells treated as in (A) and (B). (**D** to **G**) Densitometric quantification of pSPAK (D), ASC oligomers (E), GSDMD cleavage (F), and caspase-1 p20 (G) from experiments as in (C), normalized to loading controls. All data are representative of at least three independent experiments performed in duplicate or triplicate.

We next examined whether increasing extracellular potassium and chloride would exert the opposite effect. Elevation of extracellular KCl resulted in a notable, dose-dependent suppression of TcdB-induced pyrin inflammasome activation across all readouts, including ASC oligomerization, GSDMD cleavage, and caspase-1 processing (Fig. 8, A to E). Notably, high KCl strongly suppressed SPAK phosphorylation (Fig. 8, B and C), indicating inhibition of WNK1 activity under these conditions. Despite this profound suppression of inflammasome activation, pyrin dephosphorylation at Ser^205^ remained intact, demonstrating that toxin entry, processing, and upstream pyrin licensing were unaffected.

**Fig. 8.**
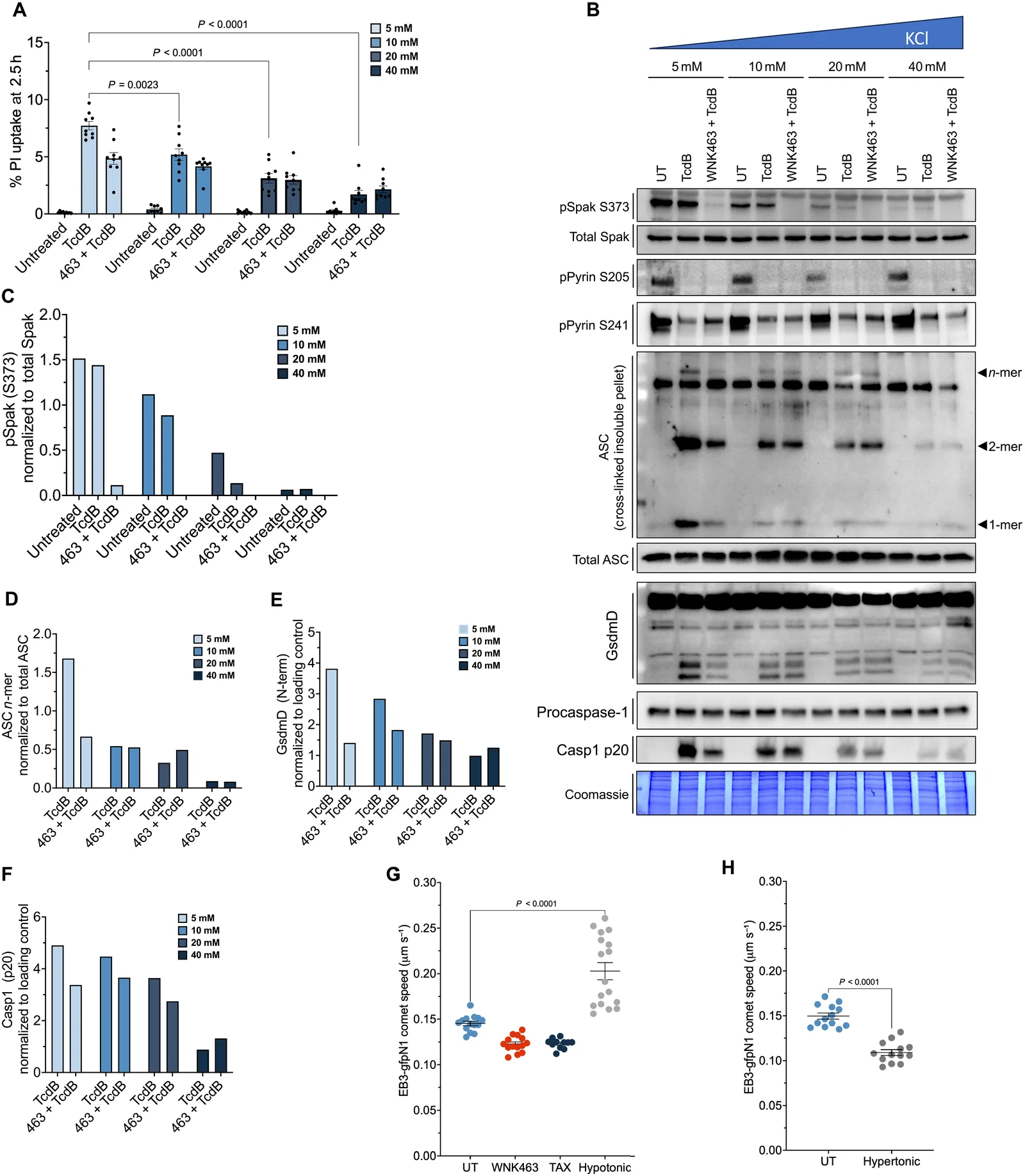
High extracellular KCl suppresses pyrin inflammasome activation and microtubule dynamics in a dose-dependent manner. (**A**) PI uptake at the time of collection of LPS-primed (500 ng ml^−1^, 8 hours) primary *Nlrp3*^−/−^ murine BMDMs pretreated with or without WNK463 (1.5 μM) in OptiMEM supplemented with KCl (final concentrations: 5, 10, 20, or 40 mM), followed by stimulation with TcdB (5 ng ml^−1^) for 2 hours and 40 min. Cells were imaged using the IncuCyte S3. Data are means ± SEM (two-way ANOVA, *n* = 9). (**B**) Immunoblots of pSPAK (S373), total SPAK, pPyrin (S205), pPyrin (S241), total pyrin, GSDMD, procaspase-1, and total ASC in cell lysates; ASC oligomers in BS^3^–cross-linked NP-40–insoluble pellets; and caspase-1 p20 in serum-free supernatants from cells treated as in (A). (**C** to **F**) Densitometric quantification of pSPAK (C), ASC oligomers (D), GSDMD cleavage (E), and caspase-1 p20 (F) from experiments as in (B), normalized to loading controls. (**G**) Quantification of EB3-EGFP comet speed (in micrometers per second) in immortalized *Nlrp3*^−/−^ macrophages treated with WNK463 (1 μM), paclitaxel (100 nM), or hypotonic OptiMEM. Data were acquired and analyzed as described in Fig. 5G. (**H**) Quantification of EB3-EGFP comet speed (in micrometers per second) in immortalized *Nlrp3*^−/−^ BMDMs cultured in OptiMEM or OptiMEM supplemented with KCl (40 mM). Data were acquired and analyzed as described in Fig. 5G.

To determine whether this inhibitory effect reflected osmotic stress per se or specific ionic regulation of WNK signaling, we compared KCl-based hypertonicity with sorbitol-induced hypertonicity. In contrast to KCl, sorbitol did not suppress pyrin inflammasome activation, despite modestly increasing SPAK phosphorylation as expected for hyperosmotic stimulation of the WNK pathway (*14*) (fig. S9). These results indicate that osmotic stress alone is insufficient to inhibit pyrin activation, whereas ion-based hypertonicity selectively suppresses pyrin inflammasome assembly by inhibiting WNK1 activity.

Collectively, these data support a model in which hypotonic conditions enhance pyrin activation by relieving chloride- and potassium-mediated inhibition of WNK1, whereas elevated extracellular KCl suppresses pyrin activation by directly inhibiting WNK1 signaling. This establishes a mechanistic link between extracellular ionic conditions and pyrin inflammasome assembly.

### Ionic conditions that modulate WNK1 activity bidirectionally regulate microtubule growth dynamics

Because extracellular potassium and chloride strongly regulated both WNK1 activity and pyrin inflammasome activation, we next asked whether these ionic conditions also influence microtubule dynamics. Using live-cell imaging of EB3-GFP–labeled microtubule plus ends, we quantified microtubule growth rates under hypotonic and KCl-enriched conditions.

Exposure of macrophages to hypotonic medium significantly increased EB3 comet velocity relative to isotonic controls, indicating enhanced microtubule polymerization dynamics (Fig. 8G). This increase contrasted with the reduced EB3 velocities observed following WNK1 inhibition or paclitaxel treatment. Conversely, elevating extracellular KCl markedly reduced EB3 comet speed, phenocopying the effects of WNK1 inhibition on microtubule dynamics (Fig. 8H).

Thus, extracellular ionic conditions that relieve chloride- and potassium-mediated inhibition of WNK1 promote microtubule growth, whereas conditions that suppress WNK1 activity are associated with reduced microtubule growth dynamics. These findings establish a tight correspondence between ionic regulation of WNK1, microtubule behavior, and pyrin inflammasome competence.

### WNK1 regulates human pyrin inflammasome in a reconstituted system

To determine whether the regulation of pyrin by WNK1 is conserved in the human pyrin inflammasome and independent of macrophage-specific signaling, we used previously described 293T cell lines stably reconstituted with human pyrin, ASC, and caspase-1 (C1AP cells) (*37*). Consistent with inflammasome specificity, TcdB robustly induced caspase-1 processing in C1AP cells. Pharmacologic inhibition of WNK1 with WNK463 markedly reduced TcdB-induced caspase-1 activation (Fig. 9, A to C). TcdB induced dephosphorylation of human pyrin irrespective of WNK1 inhibition, indicating that WNK1 functions downstream of pyrin dephosphorylation in this system.

**Fig. 9.**
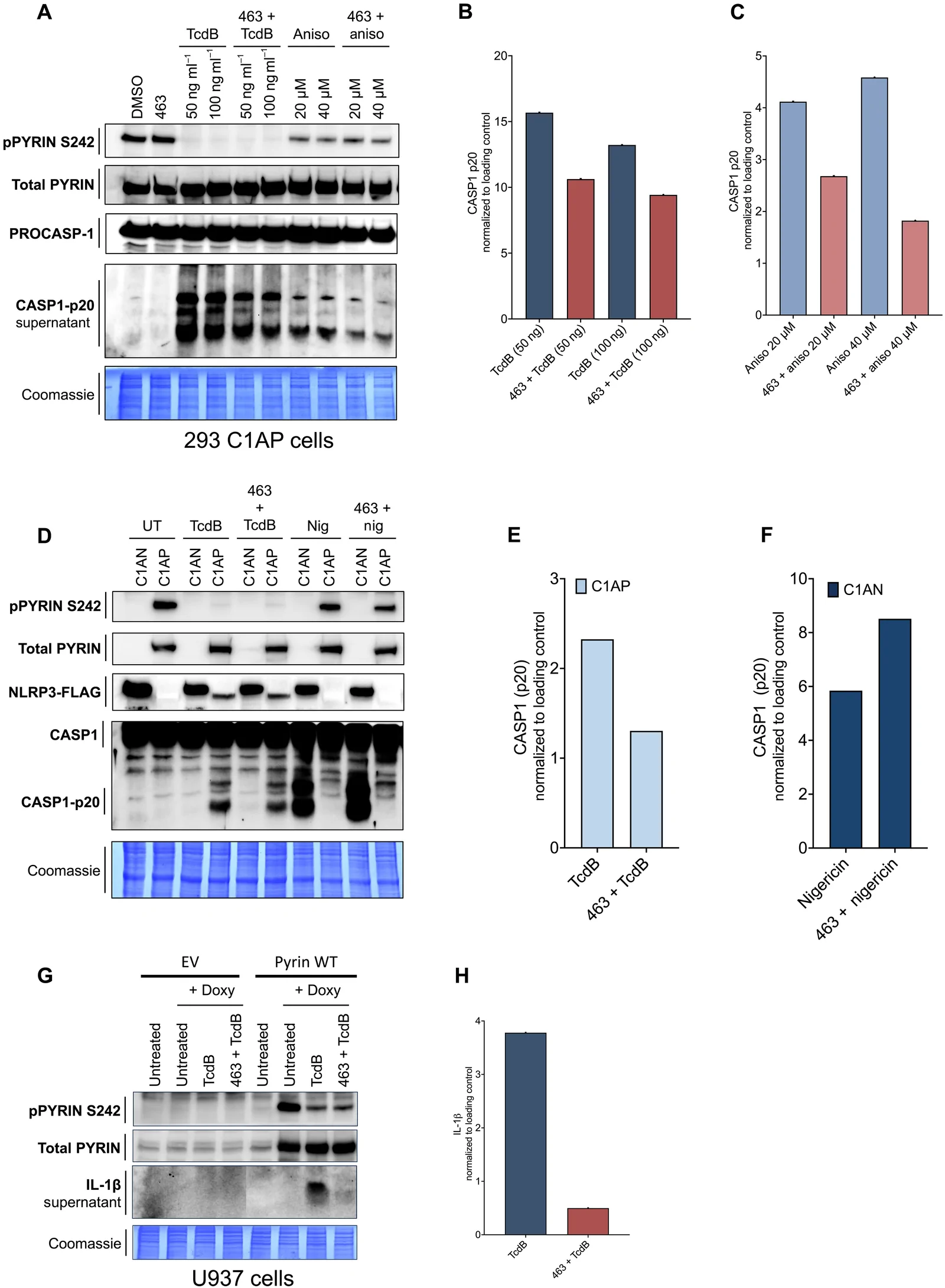
Human pyrin reconstitution systems recapitulate WNK1-dependent regulation of inflammasome activation. (**A**) Immunoblots of pPyrin (S242), total pyrin, and procaspase-1 in cell lysates; and caspase-1 p20 in culture supernatants from 293T cells stably expressing ASC, caspase-1, and pyrin (C1AP cells). Cells were pretreated with MCC950 (5 μM) with or without WNK463 (1.5 μM) for 15 min and stimulated with TcdB (50 or 100 ng ml^−1^) or anisomycin (20 or 40 μM) for 19 hours. (**B** and **C**) Densitometric quantification of caspase-1 p20/p10 cleavage products from experiments as in (A), normalized to loading controls. (**D**) Immunoblots of pPyrin (S242), total pyrin, NLRP3-FLAG, and procaspase-1 in cell lysates; and caspase-1 p20 in serum-free supernatants from 293T cells stably expressing ASC, caspase-1, and pyrin (C1AP) or ASC, caspase-1, and NLRP3 (C1AN). Cells were pretreated with MCC950 (5 μM) or WNK463 (1.5 μM) as indicated and stimulated with TcdB (100 ng ml^−1^) or nigericin (10 μM) for 7 hours. (**E** and **F**) Densitometric quantification of caspase-1 p20 cleavage in C1AP (E) and C1AN (F) cells from experiments as in (D), normalized to loading controls. (**G**) Immunoblots of pPyrin (S242) and total pyrin in cell lysates and IL-1β in culture supernatants from U937 cells expressing empty vector (EV) or human pyrin (MEFV WT). Cells were treated with phorbol 12-myristate 13-acetate (PMA) (0.1 μM) and doxycycline as indicated, pretreated with MCC950 (10 μM) with or without WNK463 (1.5 μM), and stimulated with TcdB (100 ng ml^−1^) for 4 hours. Results are representative of at least three independent experiments. (**H**) Densitometric quantification of IL-1β release from experiments as in (G), normalized to loading controls.

We next asked whether this requirement for WNK1 extends to alternative pyrin-activating stimuli. Anisomycin, which activates the human pyrin inflammasome through ribotoxic stress (*37*), induced robust pyrin dephosphorylation and caspase-1 activation. Inhibition of WNK1 with WNK463 markedly suppressed anisomycin-induced caspase-1 activation, demonstrating that the requirement for WNK1 is not restricted to RhoA-targeting toxins but reflects a general feature of pyrin inflammasome activation.

To directly compare the role of WNK1 in distinct inflammasome pathways, we analyzed parallel 293T cells stably reconstituted with human NLRP3, ASC, and caspase-1 (C1AN cells) (*37*). As expected, TcdB failed to activate caspase-1 in C1AN cells, whereas nigericin robustly induced NLRP3 inflammasome activation (Fig. 9, D to F). In contrast to its inhibitory effect on pyrin, WNK1 inhibition with WNK463 substantially enhanced nigericin-induced caspase-1 processing in C1AN cells. Thus, in a defined reconstituted system, WNK1 exerts opposing effects on pyrin and NLRP3 inflammasomes, suppressing NLRP3 while promoting pyrin activation.

To further validate these findings in a human monocytic context, we used U937 cells expressing doxycycline-inducible human pyrin (*38*). Induction of pyrin expression alone did not trigger inflammasome activation. Following doxycycline induction, TcdB stimulation induced pyrin dephosphorylation and robust IL-1β release. Consistent with our findings in mouse macrophages, WNK1 inhibition with WNK463 markedly suppressed IL-1β release without affecting pyrin dephosphorylation (Fig. 9, G and H).

Together, these results demonstrate that WNK1-dependent regulation of the pyrin inflammasome is conserved in human cells and operates independently of macrophage-specific signaling. Moreover, the opposing effects of WNK1 on pyrin and NLRP3 inflammasomes are preserved in fully reconstituted systems, indicating that this regulatory logic is intrinsic to inflammasome signaling pathways.

## DISCUSSION

Inflammasome activation is tightly controlled by upstream signaling events that ensure appropriate responses to diverse cellular stresses. Here, we identify the chloride- and potassium-sensitive kinase WNK1 as a previously unrecognized and conserved regulator of pyrin inflammasome activation. In contrast to its established role as a negative regulator of the NLRP3 inflammasome (*18*), WNK1 functions as a permissive factor required for efficient pyrin inflammasome assembly. Our findings identify WNK1 as an ion-sensitive regulator that differentially controls distinct inflammasome pathways.

A key finding of this study is that WNK1 regulates pyrin inflammasome activation largely independently of its canonical SPAK/OXSR1 signaling cascade. Although WNK1 is best known for controlling ion transport and cell volume through SPAK/OXSR1-dependent pathways (*17*), neither genetic nor pharmacological disruption of SPAK/OXSR1 signaling phenocopied the defect in pyrin inflammasome activation observed following WNK1 inhibition or deletion. Furthermore, expression of SPAK^CA^ failed to rescue pyrin inflammasome activation in WNK1-deficient macrophages. Together, these complementary approaches indicate that the canonical SPAK/OXSR1 pathway is not the principal mediator of WNK1-dependent pyrin inflammasome regulation and support the existence of an additional downstream mechanism. Although we cannot completely exclude contributions from other WNK1 substrates or compensatory signaling pathways, our data strongly support the conclusion that WNK1 regulates pyrin inflammasome activation predominantly through a noncanonical mechanism operating downstream of pyrin dephosphorylation.

These findings distinguish the regulation of pyrin from that of NLRP3, where WNK1 signaling through SPAK/OXSR1 restrains inflammasome activation by maintaining intracellular ionic homeostasis (*18*). Although SPAK^CA^ can compensate for WNK1 deficiency in developmental contexts (*39*, *40*), our results indicate that pyrin inflammasome activation is regulated largely independently of the canonical WNK1-SPAK/OXSR1 pathway, supporting a distinct noncanonical mechanism of WNK1-dependent pyrin inflammasome regulation.

Mechanistically, our data position WNK1 at the level of inflammasome assembly rather than upstream licensing (Fig. 10). WNK1 inhibition did not impair toxin entry, RhoA inactivation, or PKN1/2-dependent dephosphorylation of pyrin but instead uncoupled pyrin dephosphorylation from downstream ASC oligomerization and caspase-1 activation. Across multiple independent experiments, WNK1 inhibition did not substantially alter TcdB-induced pyrin dephosphorylation, indicating that WNK1 functions downstream of the established RhoA-PKN1/2 dephosphorylation pathway. This separation of licensing and assembly reveals an additional regulatory checkpoint in pyrin signaling and demonstrates that pyrin dephosphorylation alone is insufficient to drive productive inflammasome assembly. We also consistently observed that prolonged TcdA or TcdB stimulation reduced the abundance of endogenous pyrin. Because this decrease was most evident under conditions associated with pyrin dephosphorylation and robust inflammasome activation, it may represent a consequence of pyrin activation. The underlying mechanism remains unclear but could involve regulated protein degradation, altered biochemical solubility following inflammasome assembly, or loss of cytosolic pyrin during pyroptotic membrane rupture. Because this reduction occurred similarly in control and WNK1-inhibited cells, it does not alter our conclusion that WNK1 functions downstream of pyrin dephosphorylation.

**Fig. 10.**
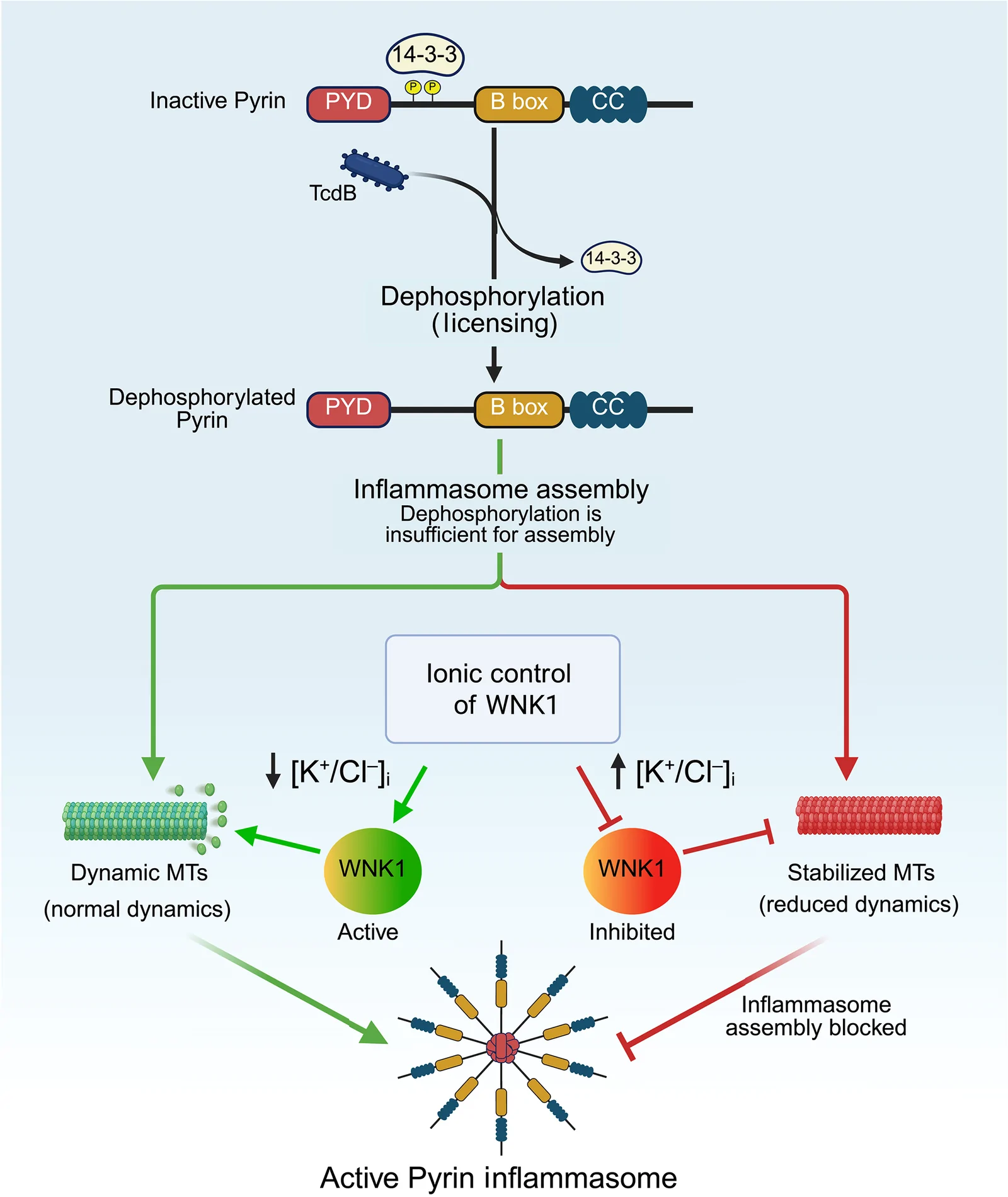
WNK1 couples ionic control to microtubule dynamics to regulate pyrin inflammasome assembly. In resting cells, pyrin is maintained in an inactive, phosphorylated state through association with 14-3-3 proteins. The bacterial toxin TcdB induces pyrin dephosphorylation, releasing 14-3-3 proteins and licensing pyrin for activation. However, pyrin dephosphorylation alone is insufficient to drive productive inflammasome assembly. Instead, assembly is regulated by the ionic control of WNK1 activity. Reduced intracellular K^+^ and Cl^−^ levels activate WNK1, promoting microtubule (MT) dynamics that are optimal for pyrin inflammasome assembly. In contrast, elevated intracellular K^+^ and Cl^−^ inhibit WNK1, resulting in microtubule stabilization and reduced dynamics, thereby impairing pyrin inflammasome assembly. Together with our pharmacological studies, these findings indicate that productive pyrin inflammasome assembly depends on appropriately regulated microtubule dynamics, whereas either microtubule hyperstabilization or depolymerization disrupts this process.

We identify microtubule dynamics as a critical downstream process regulated by WNK1. Biochemical and live-cell imaging approaches demonstrate that inhibition or loss of WNK1 stabilizes microtubules and reduces microtubule growth rates, whereas conditions that enhance WNK1 activity promote microtubule polymerization dynamics. Pharmacologic perturbation of microtubules phenocopied WNK1 inhibition, suppressing pyrin inflammasome assembly without affecting pyrin dephosphorylation. Together, these findings support a model in which WNK1 promotes the dynamic microtubule behavior required for efficient trafficking and productive assembly of pyrin inflammasome components (Fig. 10).

Recent work has identified a noncanonical role for CDC42 in promoting pyrin inflammasome assembly that is independent of its GTPase activity and does not affect pyrin dephosphorylation (*38*). This function parallels our observations for WNK1, which similarly regulates pyrin activation downstream of dephosphorylation at the level of inflammasome assembly. Notably, WNK1 has been reported to modulate Rho family GTPase pathways through SGK1-dependent regulation of CDC42 expression (*20*), suggesting a potential point of intersection between ionic signaling and cytoskeletal regulators. Although our data do not directly address whether CDC42 contributes to WNK1-dependent control of pyrin assembly, these findings raise the possibility that WNK1 and CDC42 function in parallel or convergent pathways to regulate the cytoskeletal environment required for efficient inflammasome assembly.

A central advance of this work is the identification of extracellular ionic conditions as upstream regulators of pyrin inflammasome activation through WNK1. Hypotonic stress, which reduces intracellular chloride and potassium and relieves inhibition of WNK1 (*35*, *36*), enhanced pyrin activation and microtubule growth dynamics. In contrast, elevation of extracellular KCl suppressed WNK1 signaling, reduced microtubule growth, and potently inhibited pyrin inflammasome assembly. Notably, this effect was specific to ionic conditions, as osmotic stress induced by sorbitol did not suppress pyrin activation despite activating the WNK pathway. These findings demonstrate that pyrin inflammasome assembly is selectively sensitive to ionic cues that regulate WNK1 activity, rather than to osmotic stress per se.

Hypotonic stress is known to influence multiple cellular processes, including cytoskeletal organization, vesicular trafficking, and ion homeostasis, any of which could potentially modulate inflammasome signaling independently of WNK1. Our data do not exclude contributions from these parallel responses. However, several lines of evidence support a central role for WNK1 in mediating the effects of hypotonic stress on pyrin inflammasome activation. First, hypotonic stress increased WNK pathway activity, as evidenced by enhanced SPAK phosphorylation. Second, pharmacologic inhibition or genetic deletion of WNK1 markedly attenuated the enhancement of pyrin activation under hypotonic conditions. Together, these findings support the conclusion that activation of WNK1 is a major contributor to the potentiation of pyrin inflammasome activation by hypotonic stress, while recognizing that additional WNK1-independent responses to osmotic stress may also influence this process.

These observations have important implications for understanding how different inflammasomes sense and respond to ionic perturbations. NLRP3 activation is well established to depend on potassium efflux (*12*); however, this process functions as a triggering signal rather than as a regulatory checkpoint mediated by a defined kinase. In contrast, our data indicate that pyrin inflammasome activation is modulated by potassium and chloride through WNK1, which acts as a molecular sensor linking ionic composition to cytoskeletal dynamics and inflammasome assembly. Thus, while both NLRP3 and pyrin are sensitive to ionic changes, they do so through fundamentally distinct mechanisms.

The regulatory role of WNK1 in pyrin signaling is conserved across species and cellular contexts. Using fully reconstituted human systems (*37*, *38*), we show that WNK1 inhibition suppresses pyrin activation while enhancing NLRP3 activation, recapitulating the opposing effects observed in primary mouse macrophages. These results indicate that WNK1-dependent regulation is conserved despite important structural differences between mouse and human pyrin, including the presence of the B30.2 domain in human pyrin. They further demonstrate that WNK1-dependent regulation is intrinsic to inflammasome signaling pathways and does not rely on macrophage-specific factors. Moreover, the requirement for WNK1 extends across distinct pyrin-activating stimuli, indicating that WNK1 functions as a general facilitator of pyrin inflammasome assembly. Future studies examining WNK1 signaling in peripheral blood mononuclear cells from patients with Familial Mediterranean Fever will be important to determine whether this pathway contributes to disease pathogenesis or therapeutic responsiveness.

The identification of WNK1 as an ion-sensitive regulator of pyrin inflammasome assembly raises the possibility that electrolyte disorders may influence pyrin-driven inflammation. Gitelman syndrome is characterized by chronic hypokalemia and altered chloride homeostasis, conditions known to activate WNK signaling pathways (*41*). Notably, rare reports have described the coexistence of Gitelman syndrome and Familial Mediterranean Fever (*42*, *43*), although the mechanistic basis for this association remains unclear. In this context, our findings provide a potential framework in which sustained alterations in intracellular potassium and chloride may modulate pyrin inflammasome assembly through WNK1-dependent control of microtubule dynamics. While these observations do not establish causality, they raise the possibility that ionic dysregulation may influence disease severity or penetrance in pyrin-driven autoinflammatory disorders. Further studies will be required to directly test this hypothesis.

Together, our findings support a model in which WNK1 acts as an ion-sensitive rheostat that couples intracellular ionic conditions to cytoskeletal dynamics and inflammasome competence. Under conditions of reduced intracellular chloride and potassium, WNK1 becomes activated, promoting dynamic microtubule behavior and enabling efficient pyrin inflammasome assembly. Conversely, elevated extracellular KCl suppresses WNK1 activity, stabilizes microtubules, and prevents productive inflammasome formation (Fig. 10). This framework provides a mechanistic basis for how environmental and cellular ionic fluctuations can shape innate immune responses. An important question for future investigation will be to determine how WNK1 couples ionic homeostasis to regulation of microtubule dynamics. Whether this occurs through transient interactions with cytoskeletal regulators or through currently unidentified downstream effectors remains to be determined.

In summary, our previous work demonstrated that WNK1 differentially regulates inflammasome pathways by suppressing NLRP3 and AIM2 activation while having little effect on the NLRC4, NLRP1, or noncanonical caspase-11 inflammasomes (*18*). The present study extends these findings by showing that pyrin is regulated in the opposite manner, requiring WNK1-dependent maintenance of dynamic microtubules for efficient inflammasome assembly. Together, these findings establish WNK1 as a central regulator linking ionic signals to cytoskeletal dynamics and inflammasome assembly. These results expand our understanding of pyrin inflammasome regulation and provide mechanistic insight into how ion-sensitive kinases coordinate innate immune signaling. Given the importance of pyrin in autoinflammatory diseases and host defense (*5*, *6*, *8*, *9*, *30*), targeting WNK1 or its downstream effects on the cytoskeleton may represent a promising strategy for modulating inflammasome-driven inflammation.

## MATERIALS AND METHODS

### Reagents

All reagents used in this study and their sources are listed in Table 1.

**Table 1.** Reagents and resources used in this study.

Reagents or resources	Sources	Identifier
Commercial antibodies		
Cleaved Caspase-3 (Asp^175^)	Cell Signaling Technology	Catalog no. 9664; RRID:AB_2070042
Cleaved IL-1β (Asp^116^)	Cell Signaling Technology	Catalog no. 2021; RRID:AB_2280224
Anti–phospho-Pyrin S205	Abcam	ab201784; RRID:AB_3674165
Anti–phospho-Pyrin S241	Abcam	ab200420; RRID:AB_2922814
Anti-GSDMD	Abcam	ab209845; RRID:AB_2783550
Anti-RIP3	Bio-Rad	Catalog no. AHP1797; RRID:AB_2178676
Anti-MLKL	Millipore	Catalog no. MABC604; RRID:AB_2820284
Anti–IL-1β	GeneTex	Catalog no. GTX74034; RRID:AB_378141
HRP anti-rabbit	Kwik Quant	R1006
HRP anti-mouse	Kwik Quant	R1005
HRP anti-rat	Invitrogen	31471
HRP anti-sheep	Invitrogen	31480
In-house antibodies		
Anti-Pyrin (mouse)		
Anti-Pyrin (human)		
Anti-NLRP3 (mouse)		
Anti-NLRP3 (human)		
Anti–Caspase-1 (mouse)		
Anti–Caspase-1 (human)		
Anti-ASC (mouse)		
Chemicals and recombinant proteins		
MCC950	Invivogen	MC9-40-02
WNK463	MedChemExpress	HY-100626
WNK-IN-11	MedChemExpress	HY-112094
Emricasan	MedChemExpress	HY-10396
Doxycycline hyclate	AppliChem	Catalog no. A2951
BS^3^ cross-linker	ProteoChem	c1103
Protease inhibitor cocktail	Thermo Fisher Scientific	A32955
PMA	Sigma-Aldrich	Catalog no. 524400
Phosphatase A and B	APExBIO	K1015-A and K1015-B
PMSF	Millipore	S2332
Puromycin	Invitrogen	A11138-02
Pam3CSK4	Invivogen	tlrl-pms
LPS	Sigma-Aldrich	L8274
ATP	Sigma-Aldrich	A7250
Anisomycin	Sigma-Aldrich	A9789
Bafilomycin	Sigma-Aldrich	B1793
Bumetanide	MedChemExpress	HY-17468
Colchicine	Acros Organics	AC227125000
DCPIB	Tocris	1540
DIOA	MedChemExpress	HY-137148
Hydrochlorothiazide	Sigma-Aldrich	H4749
Nigericin	Invivogen	tlrl-nig
Okadaic acid	MedChemExpress	HY-N6785
PKC412	Tocris	2992
Paclitaxel	Sigma-Aldrich	T7191
Probenecid	MedChemExpress	HY-B0545
Rafoxanide	Sigma-Aldrich	34042
VU0463271	MedChemExpress	HY-110110
d-Mannitol (mannite)	Sigma-Aldrich	M9546
d-Sorbitol	Fisher Chemical	S459-500
Potassium chloride	Fisher Chemical	P217-500
Sodium chloride	Fisher Chemical	S271-500
Potassium gluconate	Sigma-Aldrich	P1847
Choline chloride	Acros Organics	110295000
PI	Sigma-Aldrich	P4170
Commercial assay kits		
CytoTox96 LDH release kit	Promega	Catalog no. G1781
Mouse IL-1beta/IL-1F2 Quantikine ELISA Kit	R&D Systems	Catalog no. MLB00C
Active Rho Detection Kit	Cell Signaling Technology	#8820
Cell culture reagents		
DMEM	Gibco	11965-084
DMEM F12	Gibco	11320-033
OptiMEM	Gibco	31985-070
RPMI 1640	Gibco	11-875-085

### Cell culture and treatments

Primary BMDMs were harvested from the femurs of WT (C57BL/6, the Jackson Laboratory) *Aim2^−/−^ Nlrp3^−/−^* (*44*), *Nlrp3^−/−^* (*45*), *Ripk3^−/−^* (*45*), *Mlkl^−/−^* (*45*), *Wnk1/*LysM-Cre (*18*), *Spak/Oxsr1* LysM-Cre, Spak*^CA^*, and *Wnk1^−/−^* Spak*^CA^* mice and differentiated into BMDMs by culturing in Dulbecco’s modified Eagle’s medium (DMEM; Gibco, 11965-084) supplemented with 10% fetal bovine serum (FBS), 10 mM Hepes (pH 7.0) (Cytiva), and penicillin and streptomycin (100 U ml^−1^) (“complete DMEM”), and 20% L929 supernatants in 10-cm dishes at 37°C with 5% CO_2_ for 5 to 6 days. Immortalized macrophages were generated by transformation of primary BMDMs with J2-Cre retrovirus and maintained in complete DMEM. Human embryonic kidney (HEK) 293T cells expressing CASP1, ASC, NLRP3, or PYRIN were generated in (*37*) and maintained in DMEM F12 (Gibco, 11320-033) supplemented with 10% FBS, 10 mM Hepes (pH 7.0) (Cytiva), and penicillin and streptomycin (100 U ml^−1^) (“complete DMEM F12”). U937 expressing empty vector and PYRIN (MEFV-WT) were maintained in RPMI 1640 (Gibco, 11-875-085), as previously described (*38*). For various treatments, primary and immortalized BMDMs and 293T cells were seeded in 12-well plates at a density of 0.2 × 10^6^ (293T) to 0.6 × 10^6^ (BMDMs) cells per well 3 to 48 hours before treatment. On the day of experiment, BMDMs were typically primed with LPS (500 ng ml^−1^) for 3 to 8 hours, followed by stimulation with adenosine 5′-triphosphate (5 μΜ), nigericin (10 μg ml^−1^), TcdA (100 to 500 ng ml^−1^), or TcdB (5 to 250 ng ml^−1^), or in OptiMEM (Gibco, 31985-070), unless otherwise indicated. Where indicated, cells were pretreated with bafilomycin A1 (100 nM), colchicine (100 nM to 5 μΜ), emricasan (15 to 30 μΜ), MCC950 (5 to 10 μΜ), PKC412 (1 μΜ), paclitaxel (100 nM to 5 μΜ), WNK463 (1 to 1.5 μΜ) for 10 to 25 min at 37°C. For experiments involving anisomycin stimulation of HEK293T cells, cells were treated with anisomycin (20 to 40 μM) overnight. For measurement of PI uptake, 1.5 μΜ PI (Sigma-Aldrich, catalog no. P4170) was added to OptiMEM at the beginning of each experiment and plates were scanned on an IncuCyte S3 Live-Cell Analysis System and imaged at ×20 magnification under phase and red fluorescent filters. Percent PI uptake was calculated by the ratio of % red fluorescent confluence/% phase confluence × 100. For experiments involving *Casp1^−/−^* pyrin-EGFP (pyrin-gfpN1)–immortalized BMDMs, plates were scanned on the IncuCyte and imaged at ×20 magnification under phase and green fluorescent filters. Because WNK1 inhibition enhances NLRP3 and AIM2 inflammasome activation while suppressing pyrin activation, experiments using TcdB were routinely performed in the presence of MCC950 or in Aim2/Nlrp3-deficient macrophages to exclude contributions from these parallel inflammasome pathway.

### LDH release assay

LDH release in serum-free cell culture supernatants after indicated treatments was measured using the CytoTox 96 LDH release kit (Promega, catalog no. G1781) according to the manufacturer’s protocol and quantified and expressed as relative to the total LDH measured in untreated cell lysates.

### IL-1β release enzyme-linked immunosorbent assay

IL-β released into serum-free cell culture supernatants after indicated treatments was measured using the Mouse IL-1beta/IL-1F2 Quantikine ELISA (enzyme-linked immunosorbent assay) Kit (R&D Systems, catalog no. MLB00C) according to the manufacturer’s protocol and calculated on the basis of a standard curve derived from standards and reagents provided by the manufacturer.

### Immunoblot analysis

BMDMs were lysed in buffer containing 20 mM Hepes-KOH (pH 7.5), 150 mM KCl, 1.0% NP-40, protease inhibitors, and phosphatase inhibitors and clarified by centrifugation at 3825*g* for 10 min at 4°C using an Eppendorf 5417R refrigerated microcentrifuge. HEK293T cells were lysed in buffer containing 50 mM tris-HCl (pH 7.6), 150 mM NaCl, 1.0% NP-40, 0.5% sodium deoxycholate, 0.1% SDS, protease inhibitors, and phosphatase inhibitors and clarified by centrifugation at 20,000*g* for 10 min at 4°C. Protein concentrations were determined by Bradford assay, and equal amounts of total protein were resolved by SDS–polyacrylamide gel electrophoresis (PAGE) and transferred to polyvinylidene difluoride membranes (Bio-Rad). Membranes were blocked with 5% nonfat dry milk in phosphate-buffered saline (PBS) containing 0.1% Tween 20 (PBST) for 1 hour and incubated with primary antibodies overnight at 4°C. After washing, membranes were incubated with horseradish peroxidase (HRP)–conjugated secondary antibodies for 1 hour at room temperature, washed, and developed using enhanced chemiluminescence (KwikQuant) on a ChemiDoc imaging system (Bio-Rad).

For loading controls, companion SDS-PAGE gels were stained with Coomassie Brilliant Blue following electrophoresis and imaged using the ChemiDoc imaging system. Immunoblot signals were quantified by densitometry using ImageJ and normalized to the intensity of the same prominent Coomassie-stained protein band (∼75 to 100 kDa) in the corresponding lane of the companion gel. The same reference band was used for all samples within each gel unless otherwise indicated.

### ASC oligomerization assay

BMDMs were lysed in buffer containing 20 mM Hepes-KOH (pH 7.5), 150 mM KCl, 1.0% NP-40, protease inhibitors, and phosphatase inhibitors and clarified by spinning at 3825*g* for 10 min at 4°C in an Eppendorf tabletop 5417R refrigerated centrifuge. NP-40 insoluble pellets were washed 2× and subsequently resuspended in 40 μl of lysis buffer. Resuspended pellets were cross-linked with 3 mM BS^3^ (ProteoChem) for 45 min at room temperature after which 6× SDS sample buffer containing β-mercaptoethanol was added and samples were boiled for 10 min. Cross-linked pellets were then fractionated by 12% SDS-PAGE, followed by immunoblotting with ASC antibody (made in house).

### EB3-EGFP dynamics analysis by video time-lapse microscopy

Immortalized BMDMs expressing EB3-EGFP were generated by retroviral transduction using a pMSCVpuro-EB3-EGFP retroviral vector. This vector was constructed by subcloning the EB3-EGFP insert from pEGFP-N1-EB3-EGFP plasmid (*46*) (a gift from A. Akhmanova; Addgene, plasmid #190164, RRID:Addgene_190164) into the pMSCVpuro vector (Clontech). Immortalized *Nlrp3^−/−^*, *Spak^CA^*_,_ and *Wnk1^−/−^ Spak^CA^* BMDMs stably expressing EB3-EGFP were plated in MatTek 10-mm microwell 35-mm glass bottom dishes (catalog no. P35G-1.5-10-C) 1 day before treatment. Where indicated, cells were treated with WNK463 (1.5 μM), paclitaxel (150 nM), colchicine (150 nM), hypotonic medium [OptiMEM diluted 2:3 (v/v) with cell culture grade water], or hypertonic medium (OptiMEM supplemented with 40 mM KCl) for 40 min before imaging. Groups of 5 to 25 cells were imaged per independent experiment using a Nikon A1R confocal microscope equipped with a 63× oil-immersion objective. Time-lapse images were acquired for 1 min, and EB3-EGFP comet velocities were automatically tracked using Nikon Image Analysis software. Approximately 50 to 250 EB3-EGFP comets were tracked per cell, although this number varied depending on cell morphology and comet density. For each cell, the velocities of all tracked comets were averaged to generate a single mean EB3-EGFP comet velocity. Approximately 10 to 20 cells were analyzed per treatment condition across three independent experiments. Statistical comparisons were performed using the mean EB3-EGFP comet velocity of each cell as the statistical unit.

### Tubulin polymerization assay

Tubulin polymerization assay was adapted from (*33*). Briefly, cells were treated as indicated. After 1 hour of treatment, supernatant was collected and clarified by mild centrifugation (700*g*) at room temperature for 5 min. Cells were then washed twice with PBS, lysed in warm PEM [80 mM Pipes (pH 6.8), 1 mM MgCl_2_, and 1 mM EGTA] stabilization buffer (PEM base, 30% glycerol, and 0.5% Triton X-100) containing protease inhibitor cocktail, phenylmethylsulfonyl fluoride (PMSF), paclitaxel (5 μM), and GTP (1 mM) and centrifuged at 16,000*g* for 10 min, all at 37°C. The supernatant containing soluble (cytosolic) tubulin was transferred to fresh tubes, and the insoluble pellet containing polymerized (cytoskeletal) tubulin was resuspended in radioimmunoprecipitation assay lysis buffer. A portion of the cytosolic and cytoskeletal tubulin was then mixed with 2× Laemmli buffer, heated for 5 to 10 min at 95°C, and run on a 10% SDS-PAGE gel.

### Tonicity experiments

Primary BMDMs were primed with LPS (250 to 500 ng ml^−1^) for 6 to 18 hours in 20% L929 supplemented complete DMEM. Following priming, cell medium was switched to the indicated hypertonic osmolyte solutions (OptiMEM + 100 mosmol of sorbitol) or hypertonic KCl solution [OptiMEM + 100 mosmol of KCl (40 mM KCl)], or hypotonic medium [OptiMEM diluted 2:3 (w/v) with cell culture grade water) and then stimulated with TcdB in the presence or absence of WNK463.

### Measurement of active RhoA-GTP levels

Immortalized *Casp1^−/−^* pyrin-EGFP (pyrin-gfpN1) BMDMs (7.5 × 10^6^ cells) were seeded in 100-mm dishes and pretreated with MCC950 (5 μM), WNK463 (1.5 μM), or paclitaxel (1 μM) for 15 min before stimulation with TcdB (10 ng ml^−1^). RhoA-GTP levels were assessed via pull-down with Rhotekin-RBD beads using the Active Rho Detection Kit (Cell Signaling Technology, #8820) according to the manufacturer’s instructions.

### Mice

Mice were backcrossed (*N* = 6) to and maintained on a C57BL/6 background. Mouse strains were maintained under specific pathogen–free conditions at 20° to 22°C and 30 to 70% humidity on a 6 p.m./6 a.m. nocturnal dark/light cycle. Animal protocols were carried out in accordance with guidelines set forth by the Thomas Jefferson University Institutional Animal Care and Use Committee. The authors complied with all relevant ethical regulations for animal testing and research. All experiments performed in this study received previous ethical approval, and study protocols were approved by the Thomas Jefferson University Institutional Animal Care and Use Committee (approval number 01129-2). Both male and female mice (age, 2 to 4 months) were used for harvesting BMDMs.

#### *Generation of Spak*^flox/flox^; *Oxsr1*^flox/flox^; *LysMCre mice*

Homozygous LysMCre^+^ mice [B6.129P2-Lyz2tm1(cre)/fo/J mouse strain] were crossed with Spak*^flox/flox^*; Oxsr1*^flox/flox^* mice (*39*, *40*) to generate Spak*^+/flox^*; Oxsr1*^+/flox^*; LysMCre^+^ progeny. These double heterozygous mice were subsequently intercrossed to obtain Spak*^flox/flox^*; Oxsr1*^flox/flox^*; LysMCre*^+^* mice for experimental use.

#### *Generation of Spak*^CA^ *and Wnk1^−/−^; Rosa26-Spak*^CA^ *mice*

A constitutively active form of SPAK (Spak*^CA^*) was generated by site-directed mutagenesis of rat Spak cDNA to introduce phosphomimetic mutations T240E and S380E. The mutant cDNA was inserted downstream of a loxP-flanked transcriptional STOP cassette consisting of a triple polyadenylation sequence (tpA) in a PGK-neo–targeting vector and introduced into the constitutively active Rosa26 locus by homologous recombination to generate Rosa26-Spak*^CA^* mice. In the absence of Cre recombinase, the tpA cassette prevents Spak*^CA^* expression; Cre-mediated excision of the tpA cassette enables constitutive expression of Spak*^CA^*.

Because Wnk1 and Rosa26 are located on mouse chromosome 6, a recombination-based strategy was used to place both alleles in cis. Wnk1*^+/−^*; Rosa26-Spak*^CA^* mice were crossed with WT mice, and progeny were screened to identify Wnk1*^+/−^*; Rosa26-Spak*^CA^* animals in which both alleles were present on the same chromosome (cis configuration). These mice were then crossed with Wnk1*^+/−^* mice to generate Wnk1*^−/−^*; Rosa26-Spak*^CA^* offspring. This Spak*^CA^* allele functionally substitutes for WNK1 signaling in vivo, as its expression rescues the embryonic lethality associated with Wnk1 deficiency (*39*, *40*).

### Statistical analysis

GraphPad Prism software was used for statistical analysis. Unless otherwise indicated, *P* values are calculated relative to standard treatment.
