# Supplementary material for: WNK1 links ionic signals to microtubule dynamics to control pyrin inflammasome assembly
Source: Sci Adv. 2026 Oct 16;12(42):eaeh9198. doi: 10.1126/sciadv.aeh9198 (PMC13674501; doi:10.1126/sciadv.aeh9198)
Supplement: Supplementary file 1 [file sciadv.aeh9198_sm.pdf]

Science Advances
AAAS

Supplementary Materials for

WNK1 links ionic signals to microtubule dynamics to control pyrin inflammasome assembly

Genevieve Lewis *et al.*

Corresponding author: Emad S. Alnemri, emad.alnemri@jefferson.edu

Sci. Adv. **12**, eaeh9198 (2026)
DOI: 10.1126/sciadv.aeh9198

The PDF file includes:

Figs. S1 to S9
Legends for movies S1 to S3

Other Supplementary Material for this manuscript includes the following:

Movies S1 to S3

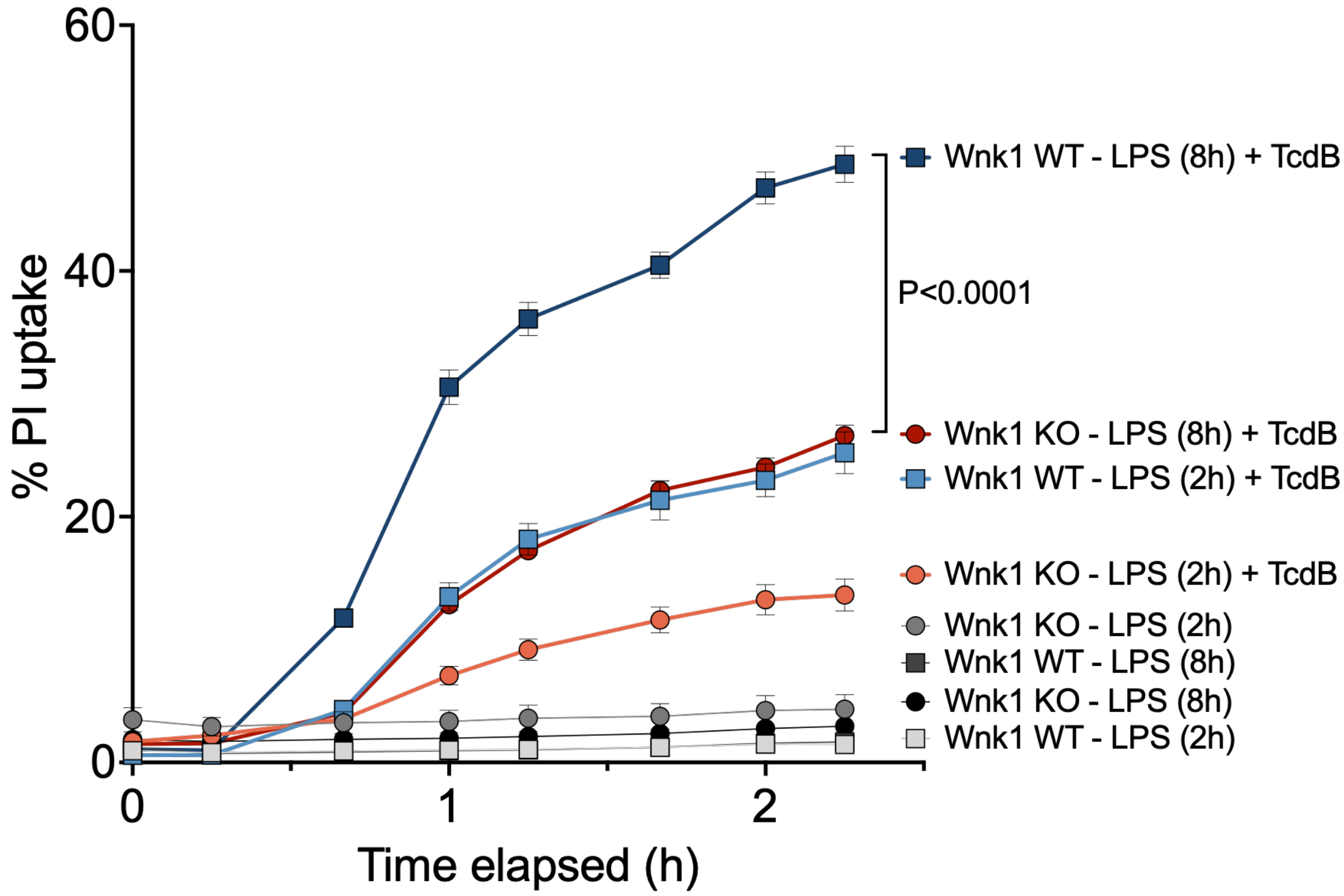

Fig. S1. WNK1 deficiency suppresses TcdB-induced pyrin inflammasome activation following both short and prolonged LPS priming.

Primary WT and Wnk1 knockout (KO) bone marrow-derived macrophages (BMDMs) were primed with LPS for either 2 h or 8 h, followed by stimulation with *Clostridioides difficile* toxin B (TcdB). Pyrin inflammasome activation was monitored by propidium iodide (PI) uptake over time using live-cell imaging. Prolonged (8 h) LPS priming enhanced TcdB-induced pyrin inflammasome activation in WT macrophages.

Importantly, Wnk1 deficiency markedly suppressed PI uptake following both short and prolonged LPS priming, demonstrating that WNK1 is required for efficient pyrin inflammasome activation following both short and prolonged LPS priming. Data are representative of four independent experiments. Error bars represent SEM.

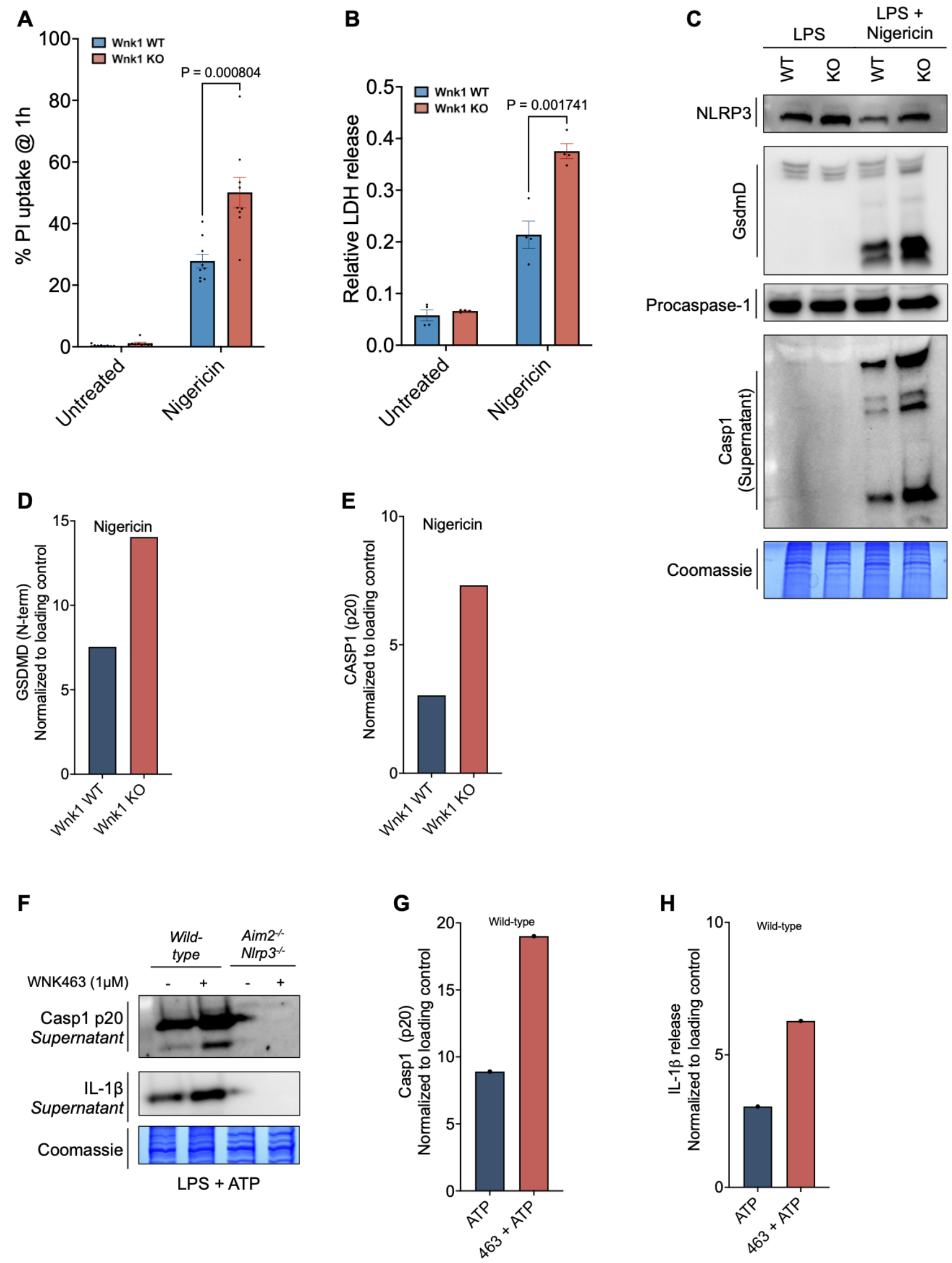

Fig. S2. WNK1 deficiency enhances NLRP3 inflammasome activation.

A, Quantification of PI uptake in LPS-primed (500 ng ml^{-1}, 3 h) primary Wnk1$^{+/+}$ LysMCre^{+} (Wnk1 WT) and Wnk1$^{fl/fl}$ LysMCre^{+} (Wnk1 KO) BMDMs treated with nigericin (10 µg ml^{-1}) for 1 h, as measured by IncuCyte S3 imaging.

B, Quantification of LDH release into the culture supernatant from the cells in (**A**), normalized to total LDH from detergent-lysed cells.

C, Immunoblot analysis of NLRP3 inflammasome activation in LPS-primed Wnk1 WT and Wnk1 KO BMDMs treated with nigericin (10 µg ml^{-1}) as in (**A**). Cell lysates were probed for NLRP3, GSDMD, and procaspase-1; culture supernatants were analyzed for caspase-1 p20.

D, **E**, Densitometric quantification of GSDMD-N (**D**) and caspase-1 p20 (**E**) bands from the immunoblots shown in (c).

F, Immunoblot analysis of NLRP3 inflammasome activation in wild-type and Aim2$^{-/-}$ Nlrp3$^{-/-}$ BMDMs stimulated with LPS and ATP in the absence or presence of WNK463 (1 µM). Culture supernatants were analyzed for caspase-1 p20 and IL-1β. Coomassie-stained gels (bottom) serve as loading controls.

G, **H**, Densitometric quantification of caspase-1 p20 (g) and IL-1β (**H**) bands from the immunoblots shown in (**F**). Data are representative of at least three independent experiments and are presented as mean ± SEM Statistical significance was determined by two-tailed unpaired t-test.

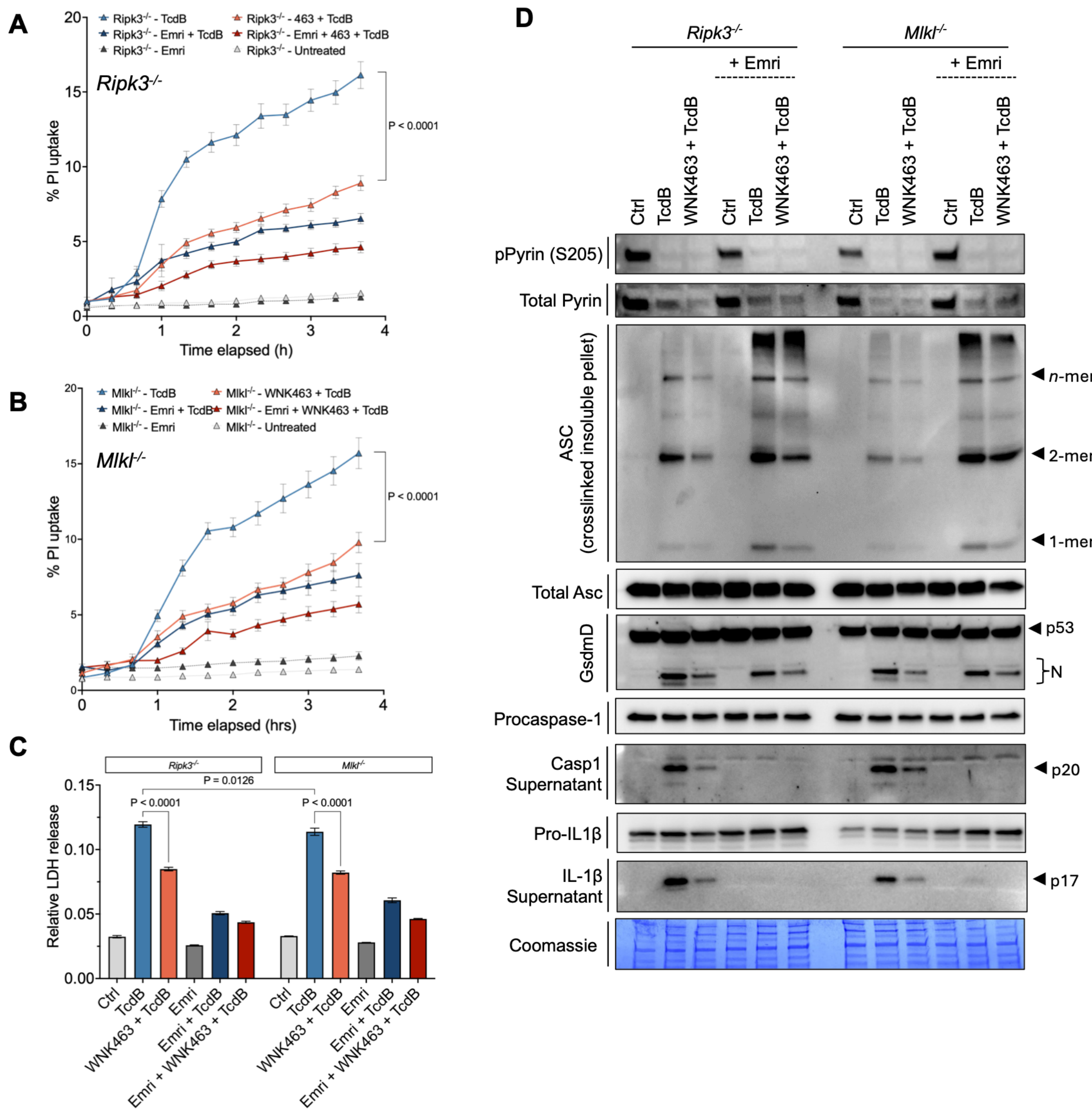

Fig. S3. WNK1 regulates pyrin inflammasome assembly independently of caspase activity and necroptotic signaling.

A, **B**, PI uptake over time in LPS-primed (500 ng ml⁻¹, 5 h) primary Ripk3⁻/⁻ and Mlkl⁻/⁻ murine bone marrow–derived macrophages (BMDMs) treated with MCC950 (5 µM) and stimulated with TcdB (5 ng ml⁻¹) in the presence or absence of WNK463 (1.5 µM) and/or the pan-caspase inhibitor emricasan (Emri, 15 µM). PI uptake was monitored using the IncuCyte S3 system.

C, Relative lactate dehydrogenase (LDH) release from LPS-primed Ripk3⁻/⁻ and Mlkl⁻/⁻ BMDMs treated as in (a), normalized to total LDH from untreated cell lysates. Error bars represent mean ± SEM.

D, Immunoblot analysis of pyrin inflammasome activation in LPS-primed Ripk3⁻/⁻ and Mlkl⁻/⁻ BMDMs treated as in (**A**). Cell lysates were probed for pPyrin (S205), total Pyrin, GSDMD, procaspase-1, and total ASC. ASC oligomers were detected in BS^3-crosslinked NP-40–insoluble fractions. Caspase-1 p20 and IL-1β were analyzed in cell culture supernatants.

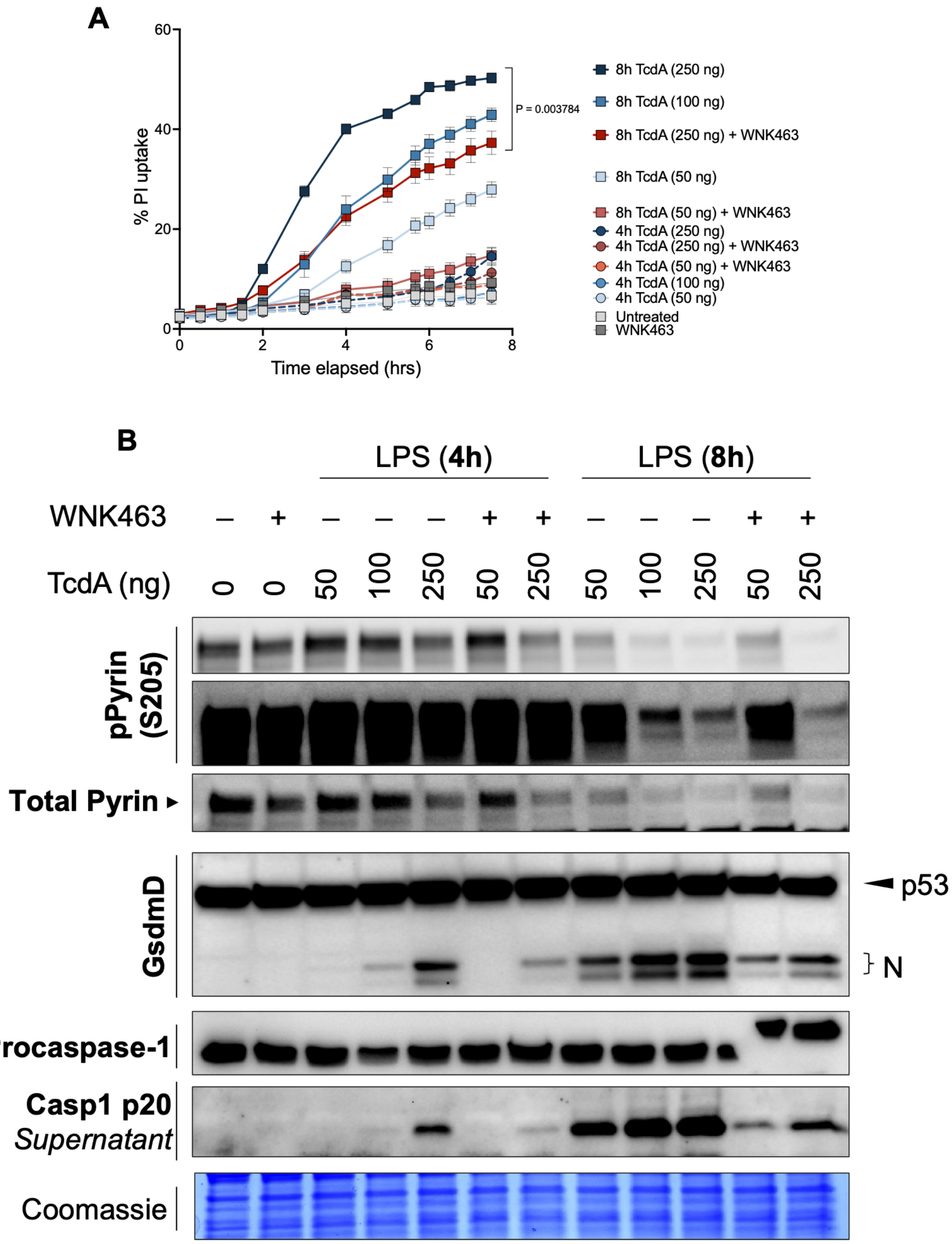

Fig. S4. WNK1 inhibition suppresses pyrin inflammasome activation induced by TcdA.

A, LPS-primed (500 ng ml⁻¹, 4 h or 8 h) primary wild-type BMDMs were pretreated with MCC950 (5 µM) in the presence or absence of the WNK1 inhibitor WNK463 (1.5 µM) for 15 min, followed by stimulation with increasing concentrations of *C. difficile* toxin A (TcdA; 50, 100, or 250 ng ml⁻¹). PI uptake was monitored over time using the IncuCyte S3 system.

B, Immunoblot analysis of pyrin inflammasome activation in cells treated as in (**A**). Cells were stimulated with the indicated concentrations of TcdA (50, 100, or 250 ng ml⁻¹) in the absence or presence of WNK463 (1.5 µM), as indicated. Shown are pPyrin (S205), total Pyrin, GSDMD, procaspase-1 in cell lysates, and caspase-1 p20 in culture supernatants. Coomassie staining of cell lysates is shown as a loading control. Data are representative of at least three independent experiments.

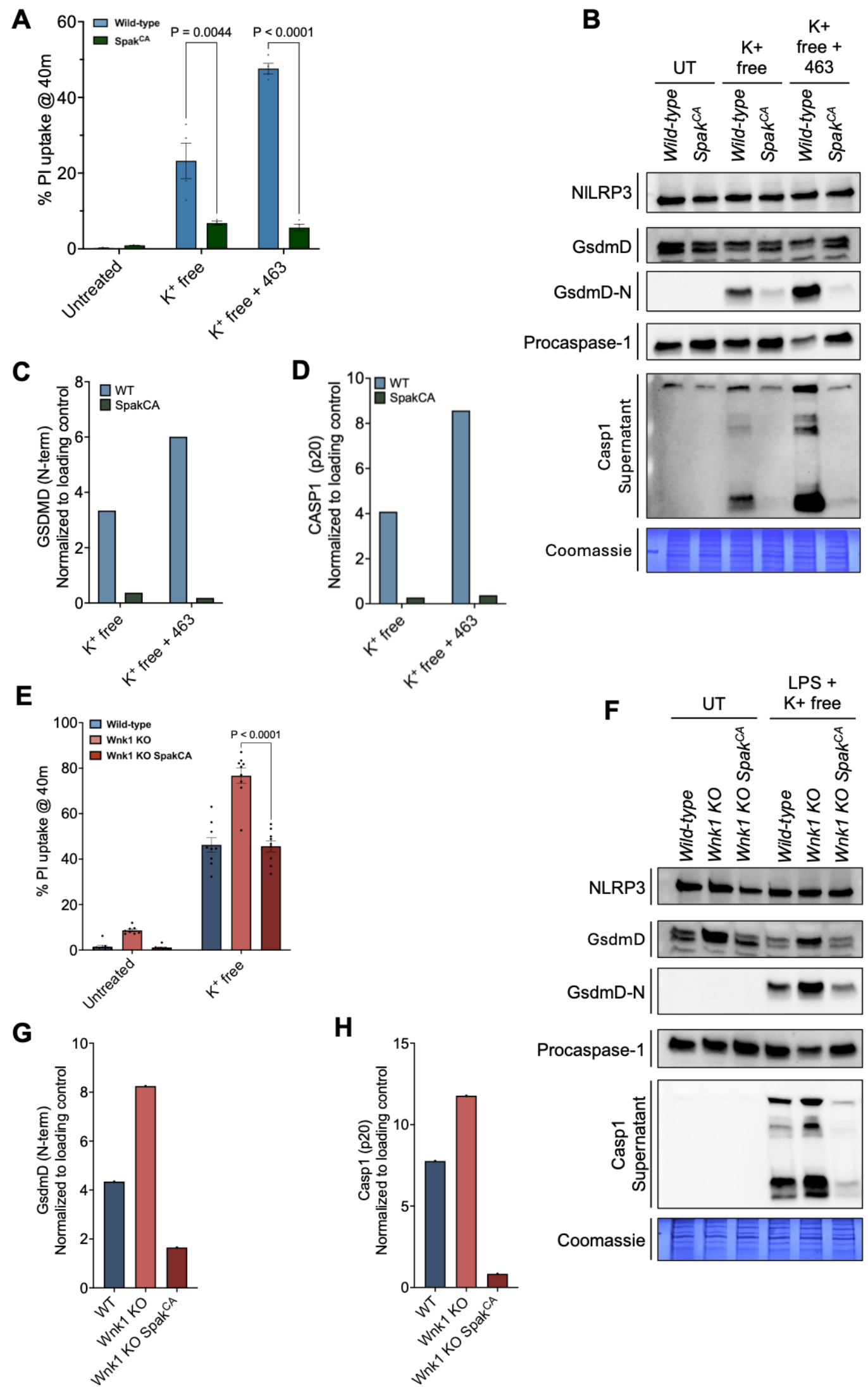

Fig. S5. Constitutive SPAK signaling suppresses NLRP3 inflammasome activation downstream of WNK1.

A, primary wild-type (WT) and Spak^{CA} BMDMs were stimulated with LPS in potassium-free medium for 40 min in the presence or absence of WNK463 (1.5 μM). Quantification of PI uptake at the time of collection, measured by IncuCyte live-cell imaging.

B, Immunoblot analysis of NLRP3 inflammasome activation in WT and Spak^{CA} BMDMs treated as in (**A**). Shown are NLRP3, GSDMD (full-length and cleaved N-terminal fragment), procaspase-1 in cell lysates, and caspase-1 p20 in supernatants. Coomassie staining is shown as a loading control.

C, D, Densitometric quantification of GSDMD cleavage (**C**) and caspase-1 p20 (**D**) from the immunoblots shown in (**B**).

E, Primary WT, Wnk1 KO, and Wnk1 KO Spak^{CA} BMDMs were stimulated with LPS in potassium-free medium for 40 min. Quantification of PI uptake at the time of collection.

F, Immunoblot analysis of NLRP3 inflammasome activation in WT, Wnk1 KO, and Wnk1 KO Spak^{CA} BMDMs treated as in (**E**), showing NLRP3, GSDMD, procaspase-1 in lysates, and caspase-1 p20 in supernatants. Coomassie staining is shown as a loading control.

G, H, Densitometric quantification of GSDMD cleavage (**G**) and caspase-1 p20 (**H**) from the immunoblots shown in (**F**).

Data are representative of at least two independent experiments performed in duplicate or triplicate.

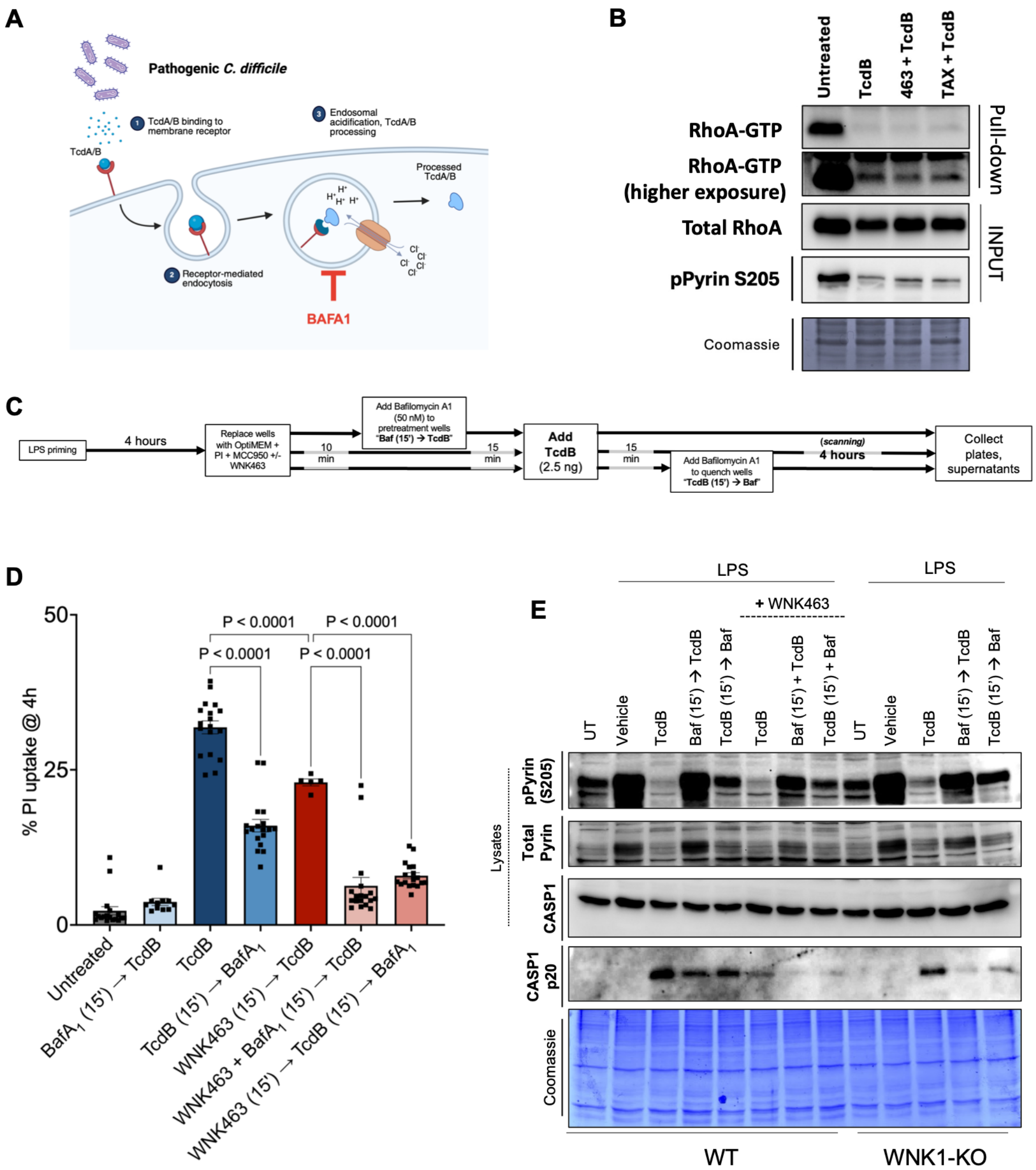

Fig. S6. WNK1 inhibition does not impair TcdB-induced RhoA inactivation or pyrin dephosphorylation, whereas inhibition of endosomal acidification blocks pyrin licensing

A, Schematic of *C. difficile* toxin B (TcdB) entry, highlighting the requirement for endosomal acidification for release of the glucosyltransferase domain.

B, Immunoblot analysis of active RhoA (RhoA–GTP) using Rhotekin-RBD pull-down assays in immortalized Casp1$^{-/-}$ BMDMs stably expressing Pyrin–EGFP. Cells were pretreated with WNK463 (1.5 μM) or paclitaxel (1 μM) in the presence of MCC950 (5 μM) for 15 min prior to stimulation with TcdB (10 ng ml^{-1}) for 3 h. RhoA–GTP levels were assessed in pull-down fractions, with total RhoA and pPyrin (S205) shown in input lysates. Coomassie staining is shown as a loading control.

C, Experimental schematic of the bafilomycin A1 (BafA1)–TcdB chase protocol used to distinguish toxin entry from downstream signaling events.

D, LPS-primed (500 ng ml^{-1}, 4 h) primary wild-type BMDMs were pretreated with MCC950 (5 μM) with or without WNK463 (1.5 μM) and/or BafA1 (100 nM) at the indicated time points relative to TcdB (5 ng ml^{-1}) stimulation. Quantification of PI uptake at 4 h, measured by IncuCyte live-cell imaging.

E, Immunoblot analysis of pPyrin (S205) and total Pyrin in cell lysates and caspase-1 p20 in supernatants from LPS-primed primary wild-type and Wnk1 KO BMDMs treated as in (**D**).

Data are representative of at least three independent experiments.

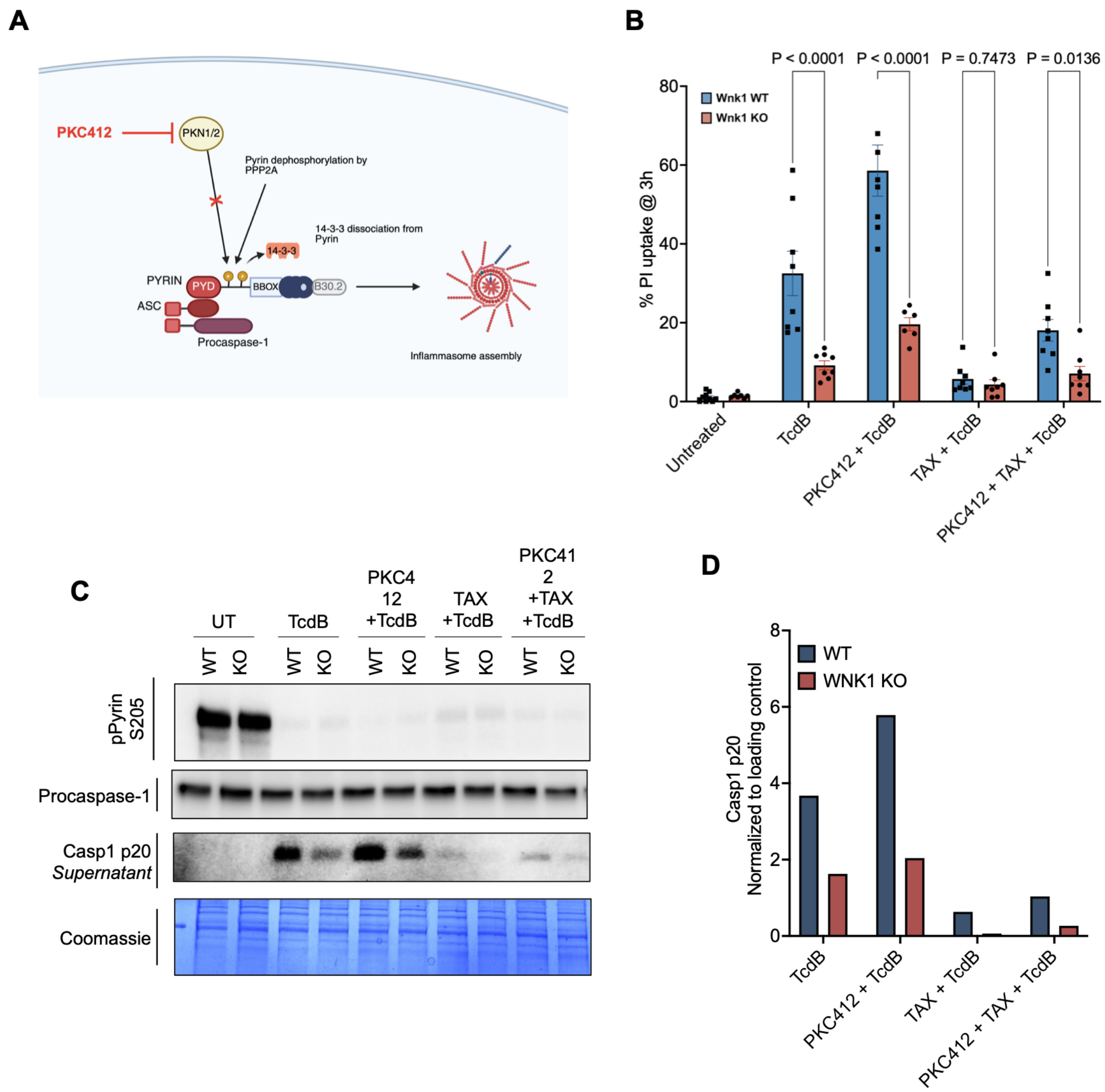

Fig. S7. Inhibition of PKN1/2 enhances pyrin activation but does not rescue the requirement for WNK1

A, Schematic illustrating regulation of pyrin phosphorylation by PKN1/2 and its pharmacologic inhibition by PKC412.

B, LPS-primed (500 ng ml^{-1}, 6 h) primary Wnk1 WT and Wnk1 KO BMDMs were pretreated with MCC950 (5 μM) with or without PKC412 (1 μM) and/or paclitaxel (150 nM) for 15 min prior to stimulation with TcdB (5 ng ml^{-1}). Quantification of PI uptake at 3 h, measured by IncuCyte live-cell imaging.

C, Immunoblot analysis of pyrin inflammasome activation in cells treated as in (**B**), showing pPyrin (S205), total Pyrin, GSDMD, procaspase-1 in cell lysates, and caspase-1 p20 in supernatants. Coomassie staining is shown as a loading control.

PKN1/2 inhibition enhanced TcdB-induced pyrin activation in wild-type macrophages but failed to restore caspase-1 activation or GSDMD cleavage in Wnk1-deficient cells. Co-treatment with paclitaxel suppressed pyrin activation under all conditions.

D, Densitometric quantification of caspase-1 p20 from the immunoblots shown in (**C**).

Data are representative of at least three independent experiments performed in duplicate or triplicate.

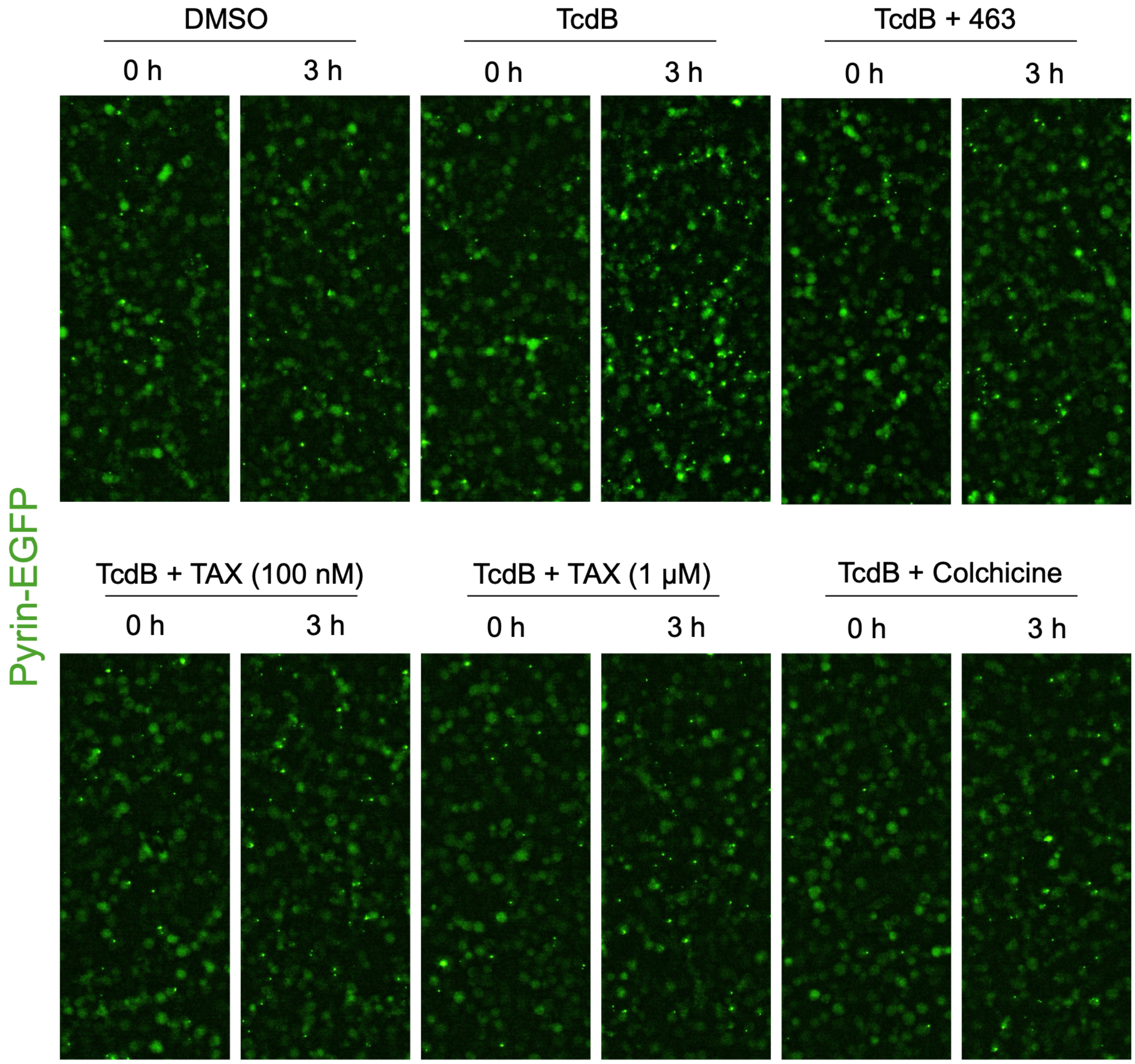

Fig. S8. Representative fluorescence images of Pyrin–EGFP oligomerization following WNK1 inhibition or disruption of microtubule dynamics. Immortalized Mefv–Pyrin-EGFP macrophages were left untreated (DMSO) or stimulated with *Clostridioides difficile*toxin B (TcdB) in the absence or presence of the WNK inhibitor WNK463 (5 μM), paclitaxel (TAX; 100 nM or 1 μM), or colchicine (1 μM). Representative fluorescence images were acquired immediately before stimulation (0 h) and after 3 h of treatment. TcdB induced robust Pyrin-EGFP speck formation, whereas WNK463 markedly suppressed pyrin oligomerization. Similarly, both microtubule stabilization with paclitaxel and microtubule depolymerization with colchicine inhibited Pyrin-EGFP speck formation, consistent with the requirement for dynamic microtubules during pyrin inflammasome assembly. Images are representative of three independent experiments.

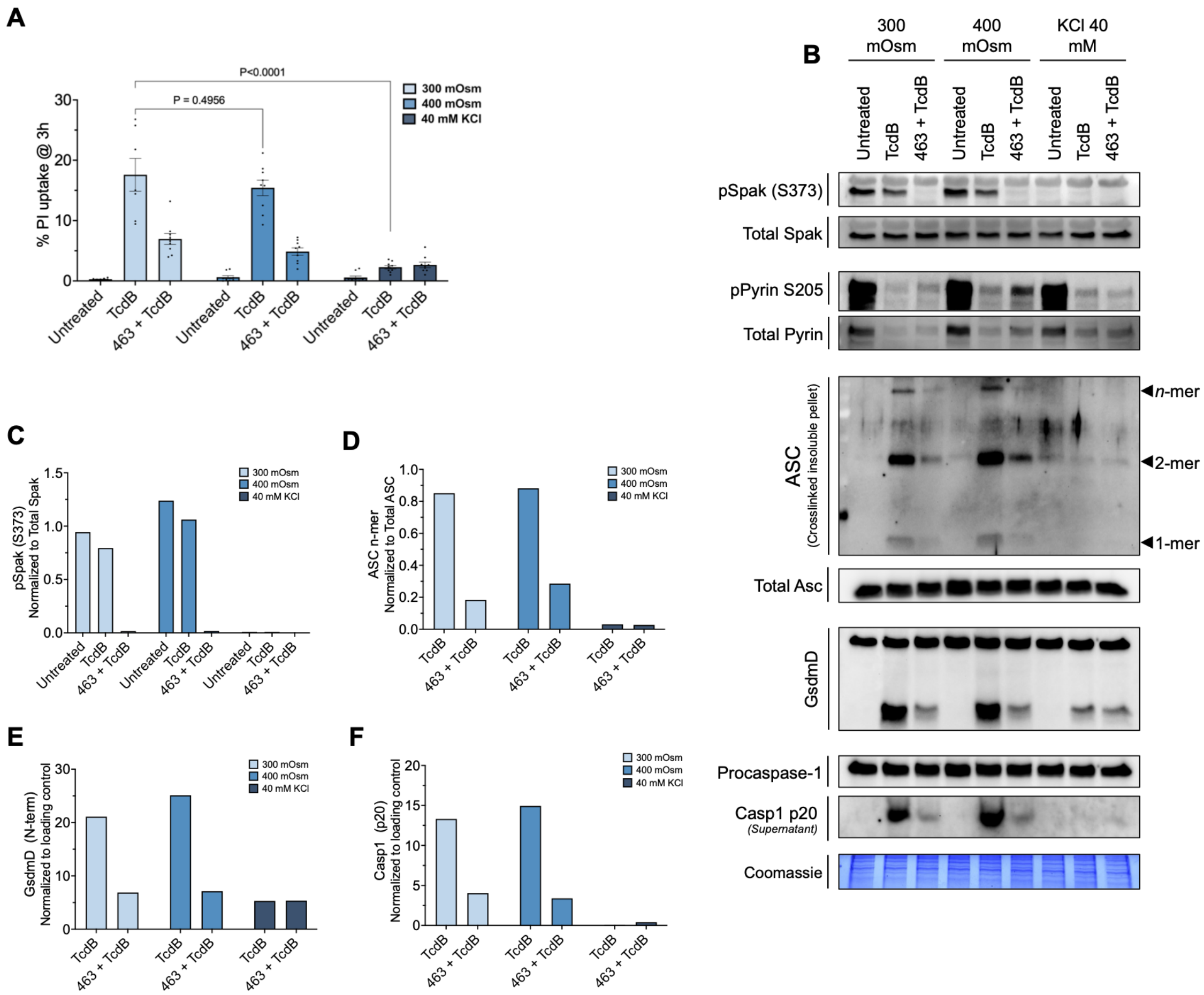

Fig. S9. Hypertonic KCl, but not sorbitol, suppresses pyrin inflammasome assembly

A, PI uptake at the time of collection of LPS-primed (500 ng ml^{-1}, 8 h) primary *Aim2*$^{-/-}$ *Nlrp3*$^{-/-}$ murine BMDMs pretreated with or without WNK463 (1.5 µM) in isotonic OptiMEM (300 mOsm), hypertonic OptiMEM supplemented with sorbitol (400 mOsm), or OptiMEM supplemented with 40 mM KCl (400 mOsm), followed by stimulation with TcdB (5 ng ml^{-1}) for 3 h. Data were acquired using the IncuCyte S3.

B, Immunoblots of pSPAK (S373), total SPAK, pPyrin (S205), total Pyrin, GSDMD, procaspase-1, and total ASC in cell lysates; ASC oligomers in BS3-crosslinked NP-40–insoluble pellets; and caspase-1 p20 in serum-free supernatants from cells treated as in (**A**).

C–F, Densitometric quantification of pSPAK (**C**), ASC oligomers (**D**), GSDMD cleavage (**E**), and caspase-1 p20 (**F**) from experiments as in (**B**), normalized to loading controls.

Data are representative of at least three independent experiments performed in duplicate or triplicate.

Supplementary movies

Movie S1. EB3-EGFP dynamics video of immortalized Nlrp3$^{-/-}$ BMDMs treated with DMSO.

Movie S2. EB3-EGFP dynamics video of immortalized Nlrp3$^{-/-}$ BMDMs treated with WNK463.

Movie S3. EB3-EGFP dynamics video of immortalized Nlrp3$^{-/-}$ BMDMs treated with paclitaxel.
